# Waste-to-sensor upcycling of polyethylene terephthalate over Ag/Zr-MOF photocatalyst for microplastic degradation and AI-assisted heavy metal detection

**DOI:** 10.1186/s13036-026-00683-4

**Published:** 2026-04-25

**Authors:** Minse Kim, Jaewon Choi, Kisung Lee, Subin Lee, Jaewon Lee, Jeong-Ann Park, Kwang Suk Lim, Suk-Jin Ha, Hyun-Ouk Kim

**Affiliations:** 1 Division of Chemical Engineering and Bioengineering, College of Art, Culture and Engineering, Kangwon National University, Chuncheon-si, Kangwon-do 24341 Republic of Korea; 2 Department of Smart Health Science and Technology, Kangwon National University, Chuncheon-si, Kangwon-do 24341 Republic of Korea; 3 Department of Mechanical and Aerospace Engineering, University of Missouri, Columbia, MO 65211 USA; 4 Department of Chemical and Biomedical Engineering, University of Missouri, Columbia, MO 65211 USA; 5 MU Materials Science and Engineering Institute, University of Missouri, Columbia, MO 65211 USA; 6 Department of Environmental Engineering, College of ACE, Kangwon National University, Chuncheon, 24341 Republic of Korea; 7 Institute of Fermentation of Brewing, Kangwon National University, Chuncheon, 24341 Republic of Korea; 8 Institute of Industrial Technology, Kangwon National University, Chuncheon, 24341 Republic of Korea

**Keywords:** Waste-to-sensor upcycling, Microplastic degradation, Ag/Zr-MOF, AI-assisted sensing

## Abstract

This study aims to develop an integrated waste to sensor platform that couples photocatalytic PET depolymerization with on stream quantification of coexisting heavy metals. We achieve this by preparing AIM-202 through surface coordination of Ag to a water stable Zr-aspartate MOF (MIP-202), creating Ag^0^/Ag^δ+^ electron sink sites that enhance charge separation and reactive oxygen species generation while preserving the parent framework. The material was characterized by FT-IR, XRD, XPS, photoluminescence, KPFM, and EPR, and its photocatalytic activity was evaluated under Xe lamp irradiation. AIM-202 (5 mg mL–1) oxidized PET with increased FT-IR oxidation indices and surface pitting, and generated soluble products that increased from 151.40 µmol (week 1) to 277.27 µmol (week 2), corresponding to 11.63% and 28.68% conversion to quantified byproducts. The PET derived fluorophore 2-hydroxyterephthalate enabled fluorescence quenching based Cu^2+^ and Fe^3+^ sensing, and physics guided models refined with lightweight machine learning provided robust concentration back calculation. The novelty lies in a closed loop design in which MOF mediated depolymerization simultaneously produces the sensing reporter and enables data assisted quantification, supporting scalable remediation and monitoring within a single integrated scheme.

**Supplementary Information:**

The online version contains supplementary material available at 10.1186/s13036-026-00683-4.

## Introduction

Microplastics are persistent pollutants in marine, freshwater, and terrestrial ecosystems owing to their inertness and thermal stability [1–4]. The beneficial characteristics of polyethylene terephthalate (PET) in packaging applications contribute to its resistance to natural degradation, resulting in the accumulation of micro- and nanoscale particles over time [5–11]. Conventional methods to remediate microplastics (e.g., mechanical filtering, high-temperature incineration, chemical oxidation, and enzymatic depolymerization) have limitations from high energy requirements, secondary waste, and low specificity. In practice, membranes foul and do not fully remove all fractions, incineration is energy-intensive and leaves residual ash, oxidation results in partial degradation, and enzymatic routes are limited by crystallinity and narrow operating windows [12, 13]. Therefore, more sustainable approaches such as photocatalysis, which can generate reactive oxygen species (ROS) in situ under mild conditions and potentially reduce stoichiometric chemical inputs and secondary waste are needed to remove microplastics with low environmental impact [14, 15].

Metal–organic frameworks (MOFs) serve as multifunctional platforms for environmental remediation and sensing because of their large surface areas, adjustable pore structures, and modular ligand properties [16–24]. Zr-based MOFs (Zr-MOFs) such as MIP-202 demonstrate considerable hydrolytic stability and offer accessible carboxylate coordination sites [25–29]. In photocatalysis, the reaction efficiency is limited by the rapid recombination of photogenerated electron–hole pairs [30, 31]. The incorporation of Ag^+^ ions establishes electron-trap sites, prolonging carrier lifetimes and enhancing reactive oxygen species (ROS) production [32, 33].

Beyond physical removal, effective water treatment [34, 35] also requires on-stream detection of co-contaminants (e.g., heavy metals and oxidation byproducts) that accompany or arise from microplastic processing [28, 36–39]. Recent advances in AI-enhanced sensing improve analysis of weak fluorescence signals in complex matrices, enabling real-time, high-sensitivity detection that surpasses threshold-based assays [40, 41]. When coupled with MOF-based photocatalytic remediation, such analytics provide an integrated workflow, leveraging the high surface area and tunable pore/chemical environments of MOFs while maintaining a heterogeneous, recoverable catalyst format that supports environmental risk reduction [42].

**Fig. 1:**
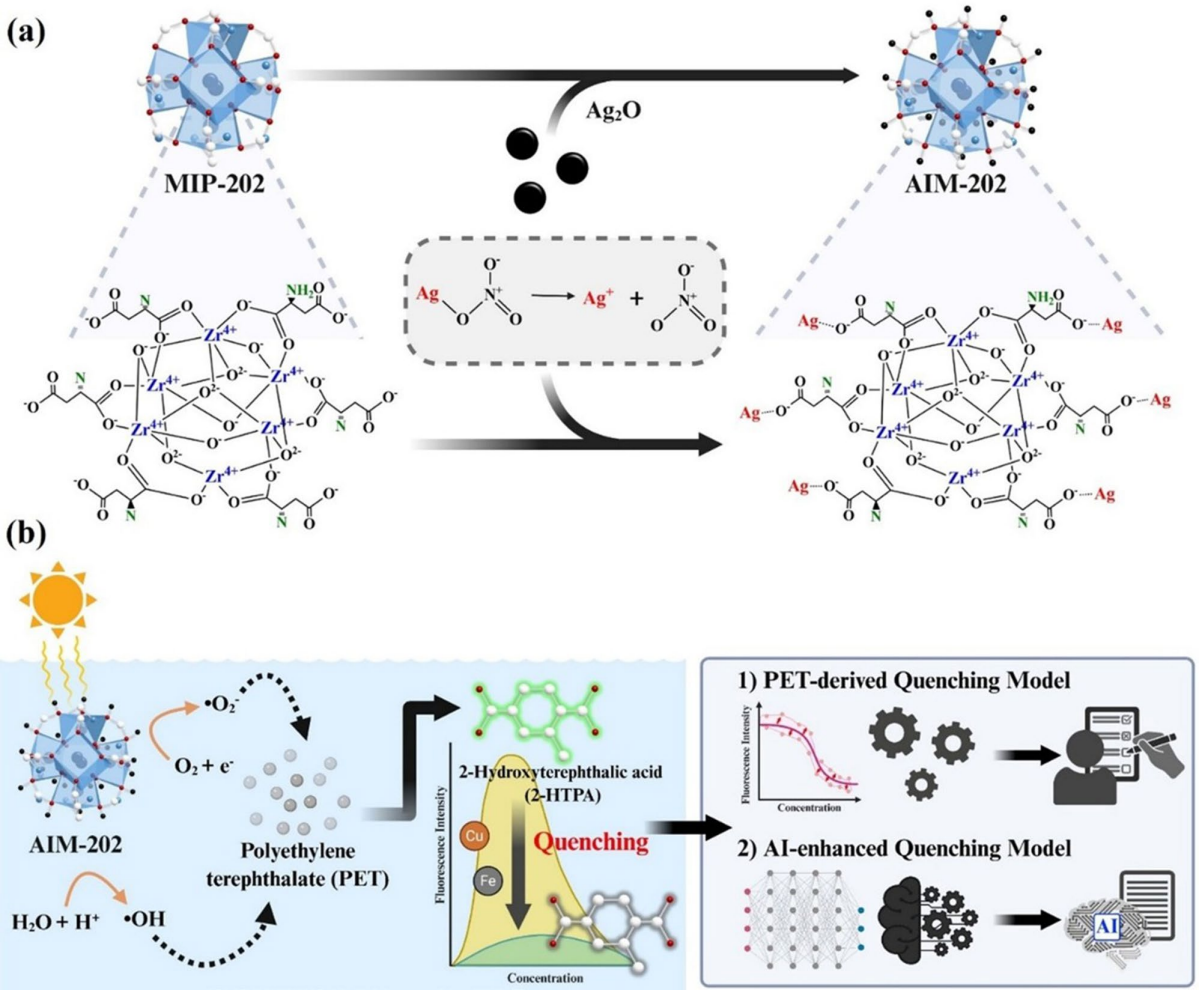
AI-enhanced waste-to-sensor upcycling of PET by AIM-202 for photocatalytic microplastic degradation and heavy metal sensing. (**a**) Ag^+^ ions coordinate to surface carboxylate groups of Zr-based MIP-202, yielding AIM-202 with enhanced charge-separation properties. (**b**) Upon UV/visible irradiation, AIM-202 generates ROS (·OH and O_2_·˗) that cleave PET microplastics into small molecules, notably 2-HTPA. The fluorescence of 2-HTPA is quenched upon binding Cu^2+^/Fe^3+^, and an AI-enhanced quenching model processes the emission data, enabling sensitive and field-deployable heavy metal detection

Herein, we report AIM-202—an Ag-doped Zr-MOF synthesized by directly coordinating Ag^+^ to surface carboxylate groups of MIP-202. AIM-202 is used to evaluate PET microplastics photodegradation to soluble aromatic byproducts under irradiation. Furthermore, it enables fluorescence-based detection of heavy metal ions via photolysis product 2-hydroxyterephthalic acid (2-HTPA), which emits near 450 nm and undergoes selective quenching by Cu^2+^ and Fe^3+^ [43–46]. Using this quenching process and AI-enhanced analytical models, we assess detection at low concentrations in Cu^2+^/Fe^3+^ solutions. Such an integrated trash-to-sensor approach provides a cohesive framework for simultaneous microplastic remediation and heavy metal monitoring. We outline a path toward decentralized PET remediation under low-intensity light and onsite Cu^2+^/Fe^3+^ monitoring using simple optics or smartphone readouts. Additionally, modular coordination chemistry and model retraining enable extension to other plastics and metal targets (Fig. 1).

## Methods

### Materials

Zirconium(IV) chloride, L-aspartic acid, silver nitrate, acetonitrile, PET, sodium hydroxide, copper(II) nitrate trihydrate, iron(III) chloride hexahydrate, 2,2,6,6-tetramethylpiperidine (TEMP), and dimethyl sulfoxide were purchased from Sigma-Aldrich (St. Louis, MO, USA). 5,5-dimethyl-1-pyrroline N-oxide (DMPO) was purchased from Supelco, Inc. (Bellefonte, PA, USA). Ethanol was purchased from Duksan Pharmaceutical Co. (Seoul, Korea).

### Synthesis of MIP-202

MIP-202 was synthesized using a hydrothermal method. Specifically, 42 mmol of L-aspartic acid was dissolved in 10 mL of distilled water (DW) under sonication to produce a clear solution. Then, 20 mmol of zirconium(IV) chloride (ZrCl_4_) was gradually added under continuous stirring. Another 10 mL of DW was added, and the mixture was stirred at 400 rpm until it became transparent. The solution was sonicated for 3 min to ensure complete homogenization. Subsequently, 10 mL of DW was added, and the reaction mixture was stirred at 400 rpm while being heated at 120 °C for 12 h using a heating mantle. The resulting solid was rinsed thrice with a 1:1 (v/v) mixture of DW and ethanol, followed by lyophilization for 24 h to obtain dry MIP-202 powder.

### Synthesis of AIM-202

To prepare AIM-202, 0.177 mmol of silver nitrate (AgNO_3_) was completely dissolved in 8 mL of acetonitrile under sonication. Subsequently, 65 mg of the pre-synthesized MIP-202 was added, and the mixture was stirred at 1,200 rpm for 4 h at room temperature. This doped suspension was washed with a 1:1 (v/v) mixture of DW and ethanol to remove the unreacted species. The final product was collected and lyophilized to obtain AIM-202.

### Characterization of MIP-202 and AIM-202

**Fig. 2:**
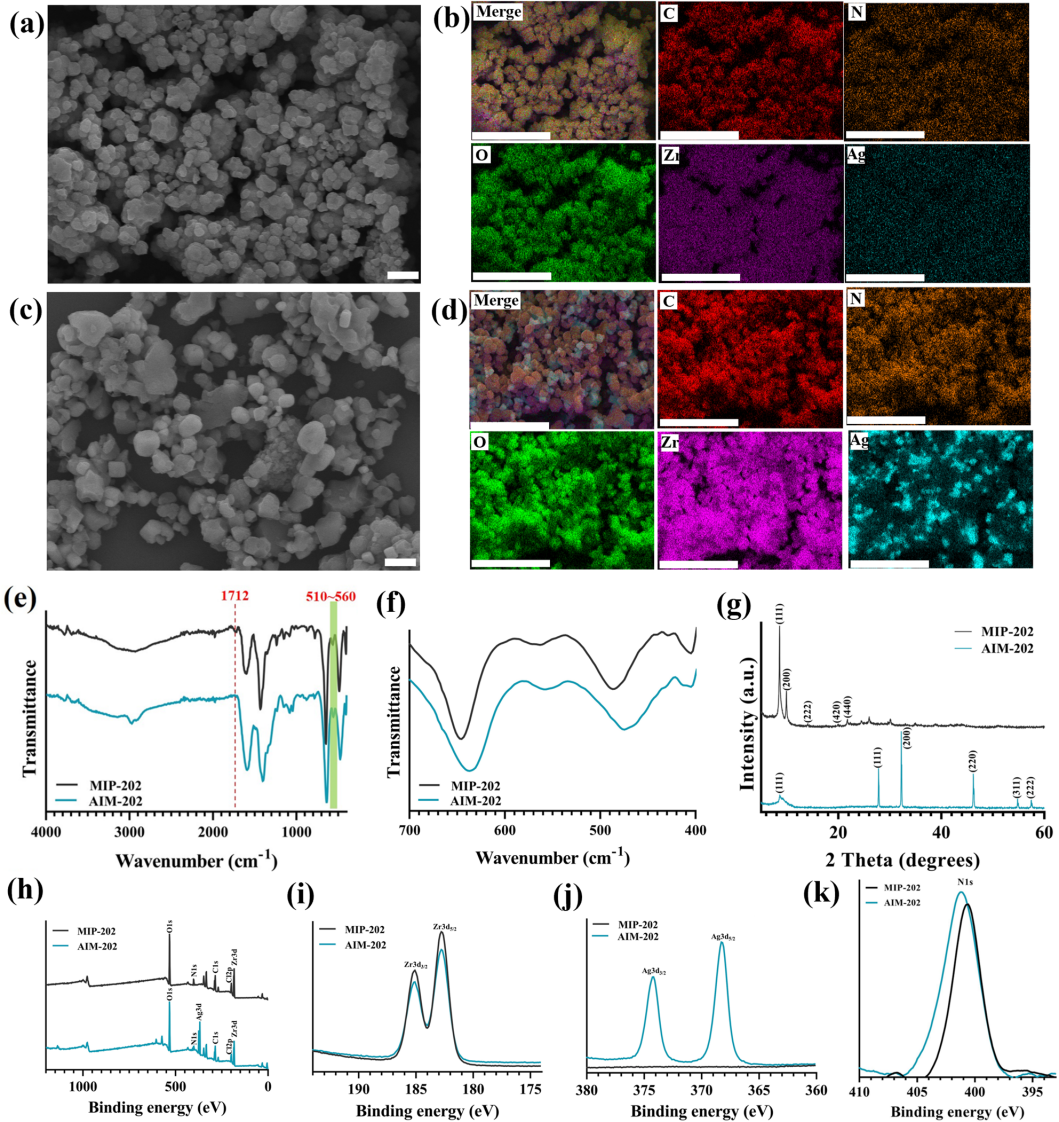
Structural and elemental characterization of AIM-202 (Ag-doped MIP-202) and MIP-202. (**a**,** b**) SEM images of (**a**) MIP-202 and (**b**) AIM-202, showing retention of the polyhedral morphology of the parent MOF after Ag coordination. Scale bars: 1 μm. (**c**,** d**) EDS elemental maps of (**c**) MIP-202 and (**d**) AIM-202, showing homogeneous distributions of C (red), N (orange), O (green), and Zr (magenta), with a distinct Ag signal (cyan) present only in AIM-202. Scale bars: 5 μm. (**e**) FT-IR spectra, showing conserved linker vibrations and a redshifted C = O stretching band near 1712 cm^−1^ for AIM-202. (**f**) Low-frequency FT-IR spectra, revealing a new band at 510–560 cm^−1^ assigned to Ag–O/Ag–O–C modes. (**g**) Powder XRD patterns. AIM-202 retained the framework reflections of MIP-202, while additional peaks at 38.1°, 44.3°, 64.4°, 77.4°, and 81.5° were indexed to fcc Ag (111), (200), (220), (311), and (222), respectively. No additional peaks indicative of crystalline impurities were detected beyond the MOF framework and the fcc Ag domains. (**h**) XPS survey spectra, verifying the presence of C, N, O, and Zr in both samples and Ag only in AIM-202. (**i**) Zr 3d core level XPS spectra of MIP-202 and AIM-202, indicating the presence of intact Zr^4+^ nodes. (**j**) Ag 3d core level XPS spectra, showing a doublet at ~ 368.2 and ~ 374.2 eV with a ~ 6.0 eV splitting, consistent with the presence of surface Ag⁰/Agδ^+^ anchored to the MOF. (**k**) The N 1s region confirms the presence of nitrogen species within the framework

Unless otherwise noted, measurements were performed at 25 °C and ambient humidity. The particle size distributions of MIP-202 and AIM-202 were analyzed via DLS and zeta-potential measurements (ELSZ-2000 S, Otsuka Electronics Korea Co., Ltd., Seongnam-si, Republic of Korea). MIP-202 and AIM-202 powders were sonicated for 3 min with a concentration of 1 mg mL^−1^ in DW immediately before measurement, for dispersion. The morphology was examined using field-emission scanning electron microscopy (FE-SEM; JSM-7900 F, JEOL Ltd., Tokyo), and the elemental composition was analyzed via EDS (Oxford Instruments, UK) using the SEM instrument. The dispersed MIP-202 and AIM-202 samples were dried on Si wafers and then coated with Pt via plasma sputtering. The high-resolution morphology and elemental compositions were further investigated using a field-emission transmission electron microscopy (FE-TEM) system at 200 kV (JEM-2100 F, JEOL Ltd., Tokyo, Japan) equipped with an EDS detector (Aztec, Oxford Instruments, UK). Dispersions of 0.1 mg mL^−1^ in DW were drop-cast onto Cu grids and dried. The surface functional groups were identified through FT-IR spectroscopy (ALPHA II, Bruker, Germany). Spectra were recorded in the attenuated total reflectance (ATR) mode with a diamond crystal. Baseline correction and normalization to 1410 cm^−1^ were applied. Diagnostic bands were assigned at 1712, 1241, 1097, and 725 cm^−1^, as described in Fig. 2. The crystalline structures were analyzed using XRD (D8 ADVANCE, Bruker AXS GmbH, Germany), and the surface elemental states were investigated via XPS (K-Alpha+, Thermo Fisher Scientific, UK). Powder XRD analysis was performed under the following conditions: Cu radiation (maximum voltage: 50 kV / maximum power: 5.4 kW); focal brightness, 6 kW/mm^2^; line focus, 0.3 × 3 mm^2^. XPS was performed with an Al anode under ultrahigh vacuum (≤ 5 × 10˗^9^ mbar), and high resolution N 1s spectra were additionally collected and analyzed to elucidate the N coordination environment associated with the Zr–aspartate network.

The fluorescence spectra of MIP-202 and AIM-202 were recorded under both dark and illuminated conditions using a microplate reader (SpectraMax i3x, Molecular Devices, CA, USA). KPFM and atomic force microscopy (AFM) were performed using an FX40 system (Park Systems, Korea) to examine the surface topography, height profile, and potential distribution of the samples. AFM topography (tapping) and KPFM were performed with the electrostatic force microscopy head operating in the amplitude-modulation mode.

### Evaluation of photogenerated charge behavior and ROS generation

EPR spectroscopy was performed to evaluate the ROS generated from MIP-202 and AIM-202 upon light irradiation. To detect the hydroxyl radical (•OH), a 1 mg/mL aqueous dispersion of each MOF was added with 25 mM DMPO. For singlet oxygen (^1^O_2_) detection, 25 mM TEMP was used under identical conditions.

Each sample was divided into two groups: one was incubated at room temperature for 5 min in the dark (control), and the other was irradiated with a handheld LED light source (365 nm, UVA) for 5 min. After treatment, all suspensions were filtered through a 0.8-µm membrane filter and then immediately analyzed using an X-band EPR spectrometer (JES-X320, JEOL Ltd., Japan).

### PET degradation using AIM-202

AIM-202 was dispersed in 40 mL of DW, and 2 g of PET granules with a diameter of approximately 4 mm were added to the dispersion. The mixture was stirred and irradiated using a Xe lamp (300 W, output wavelength: 300–1100 nm, DY Tech, Korea) to initiate photodegradation. After a specific amount of time, the supernatant containing degradation products was collected, filtered through a 0.8-µm membrane, and subsequently lyophilized. The remaining PET granules that were not degraded were recovered and thoroughly washed with DW. To remove the residual organic intermediates, the recovered PET was immersed in an aqueous NaOH solution (pH 12.5) and stirred at 1200 rpm for 3 h. This PET was washed again with DW, collected, and dried in a desiccator.

### Characterization of PET after photodegradation

Multiple analytical techniques were employed to evaluate the structural and chemical changes in PET after photocatalytic degradation by AIM-202. The surface morphology was analyzed using FE-SEM. Before imaging, the samples were sputter-coated with Pt to increase the surface conductivity. To examine alterations in the functional groups using FT-IR spectroscopy, dried PET samples were analyzed in the ATR mode, and distinctive peaks associated with ester, ether, aromatic, and carbonyl groups were identified and compared to those of untreated PET.

For product analysis, the supernatant after filtration was lyophilized and reconstituted in dimethyl sulfoxide (DMSO)-d₆. Maleic acid (0.2 mg/mL) was incorporated as an internal standard. This solution was examined via 1 H NMR spectroscopy (Bruker Avance Neo 600, Bruker, Germany) to measure the production of TPA and EG as characteristic degradation products. The peak integrals of the aromatic and methylene protons were compared to those of the internal standard to determine their concentrations.

### Fluorescence-based detection of heavy metal ions using PET-derived 2-HTPA

The fluorescence sample originated from the 2-week PET photodegradation run performed with AIM-202 (5 mg mL^−1^) under a Xe lamp, as shown in Fig. 4 and described in Sect.  4.6. For product analysis, the supernatant after filtration was lyophilized. The lyophilized powder was reconstituted in a mixed solution of 100 µL of DMSO and 900 µL of DW to prepare the 2-HTPA solution for fluorescence examination. Copper(II) nitrate trihydrate and iron(III) chloride hexahydrate were each dissolved in DW to prepare Cu^2+^ and Fe^3+^ solutions at different concentrations. Equal volumes of the 2-HTPA solution and each metal ion solution were combined to create sensing samples with various ion concentrations.

Aliquots of each mixture (100 µL) were placed in separate wells of a black 96-well microplate. The samples were incubated for 10 min at 25 °C in the absence of light to facilitate interactions between the fluorophore and metal ions. Then, the fluorescence intensity was quantified using a microplate reader (SpectraMax i3x, Molecular Devices, USA) with excitation at 370 nm and emission at 470 nm. All measurements were conducted in triplicate to ensure repeatability.

### Data analysis and modeling

All fluorescence-quenching data were analyzed using Python 3.10 within the Jupyter Notebook environment. Experimental datasets were analyzed using Pandas, and numerical computations were executed with NumPy. Data visualization was conducted using the matplotlib.pyplot module.

Nonlinear regression was conducted for curve fitting of the mechanistic models, including the Langmuir and Stern–Volmer equations, utilizing the curve_fit function from the scipy.optimize module.

To enhance the predictive accuracy and model adaptability, regression models based on machine learning were executed using the scikit-learn toolkit. For Cu^2+^ detection, 7 concentration levels (0, 2.5, 5, 15, 20, 30, 40 µM; 7 mean points) were used, and an RBF SVR residual model (C = 1e5, gamma = 0.05, epsilon = 0.1) was trained to correct the residuals of the PET derived Langmuir quadratic baseline. Hyperparameters C, γ, and ε were meticulously adjusted to reduce the prediction error. For Fe^3+^ detection, 7 concentration levels (0, 1, 2.5, 10, 20, 50, 100 µM; 7 mean points) were used, and a second-order polynomial residual correction model (PolynomialFeatures, degree = 2; LinearRegression) was trained to correct the residuals of the PET-derived logarithmic baseline.

Model performance was assessed using R^2^ and RMSE with LOOCV (6/7 training, 1/7 test per fold); LOOCV performance was Cu^2+^ R^2^ = 0.9494 and RMSE = 29,865.31 a.u., and Fe^3+^ R2 = 0.9034 and RMSE = 50,819.39 a.u., while the final calibration curves in Fig. 6 were obtained by refitting the selected models on the full datasets (Cu^2+^ R2 = 0.9999995, RMSE = 94.01 a.u.; Fe^3+^ R2 = 0.9962, RMSE = 10,113.63 a.u.), computed using the r2_score and mean_squared_error functions, respectively, from the scikit-learn.metrics module. Model fitting and residual visualization were performed using conventional charting methods in matplotlib. All model implementations, including parameter extraction, training, and prediction, were executed in a modular and reproducible manner using Jupyter Notebook cells.

## Results and discussion

### Surface Ag coordination with preserved Zr-MOF architecture

Previous Zr-MOF photocatalysts relied on bulk substitution or nanoparticle deposition, often disturbing the framework and reducing porosity. Evidence of lattice preservation was indirect. In contrast, our surface-confined Ag coordination preserves crystallinity and creates Ag⁰/Agδ^+^ electron-sink sites. AIM-202 was synthesized by coordinating Ag^+^ to carboxylate and O sites on the MIP-202 surface, localizing Ag at the exterior. SEM revealed AIM-202 retained the polyhedral morphology of MIP-202 (Fig. 2(a, c)). Elemental mapping confirmed uniform C, N, O, Zr, with Ag only in AIM-202 (Fig. 2(b, d) and Table S1). These observations suggest that Ag incorporation occurred via coordination instead of lattice substitution. Transmission electron microscopy–energy-dispersive X-ray spectroscopy (TEM–EDS) confirmed Ag localization in AIM-202 (Fig. S1 and Table S2). These data support lattice preservation with surface electron-sink sites.

Colloidal measurements can distinguish between surface and bulk doping. Dynamic light scattering (DLS) revealed a modest increase for AIM-202 (Fig. S2(a)). The zeta-potential shifted from + 19.8 mV (MIP-202) to − 1.8 mV (AIM-202), consistent with charge neutralization and interfacial band bending (Fig. S2(b)).

Surface-coordinated Ag(I) has high electron affinity and efficiently captures photogenerated electrons, thereby reducing electron–hole recombination and prolonging carrier lifetimes at the solid–liquid interface. The negative surface potential promotes charge separation and ROS formation (•OH, ^1^O_2_).

Ag coordination that is confined to the linker O atoms perturbs the local ligand field, while preserving the Zr-MOF lattice. Fourier transform infrared (FT-IR) spectroscopy revealed L-aspartate features with a carbonyl redshift (from ~ 1720 to ~ 1712 cm^−1^), indicating electron withdrawal upon Ag binding (Fig. 2(e)). AIM-202 also exhibited a new weak band at 510–560 cm^−1^ (Fig. 2(f)) assignable to the Ag–O/Ag–O–C modes, supporting an interfacial coordination. The crystallinity of the framework was retained, whereas Ag nucleated as nanocrystalline surface domains. X-ray diffraction (XRD) revealed preserved MIP-202 reflections with new face-centered cubic (fcc)-Ag peaks (2θ ≈ 38.1°, 44.3°, 64.4°, 77.4°, 81.5°) (Fig. 2(g)). These Ag reflections are consistent with nanocrystalline fcc Ag formed on the MOF surface, and no additional diffraction peaks attributable to other crystalline impurity phases were observed.

XPS detected Ag only in AIM-202 (Fig. 2(h)), while the Zr 3d peak remained at ~ 182.4/184.7 eV, confirming intact Zr^4+^ nodes (Fig. 2(i)). The Ag 3d doublet at ~ 368.2/374.2 eV with ~ 6.0-eV spin–orbit splitting (Fig. 2(j)) is characteristic of metallic Ag⁰ with weakly coordinated Agδ^+^. Taken together with the FT-IR and XRD results, these data indicate that interfacial Ag is partially reduced yet bound to O donors, providing two complementary electron-capture pathways. Furthermore, the biological safety of the material was assessed via in vitro cytotoxicity assays, which confirmed high cell viability (> 90%) for AIM-202 exposures up to 4 h (Fig. S12).

**Fig. 3:**
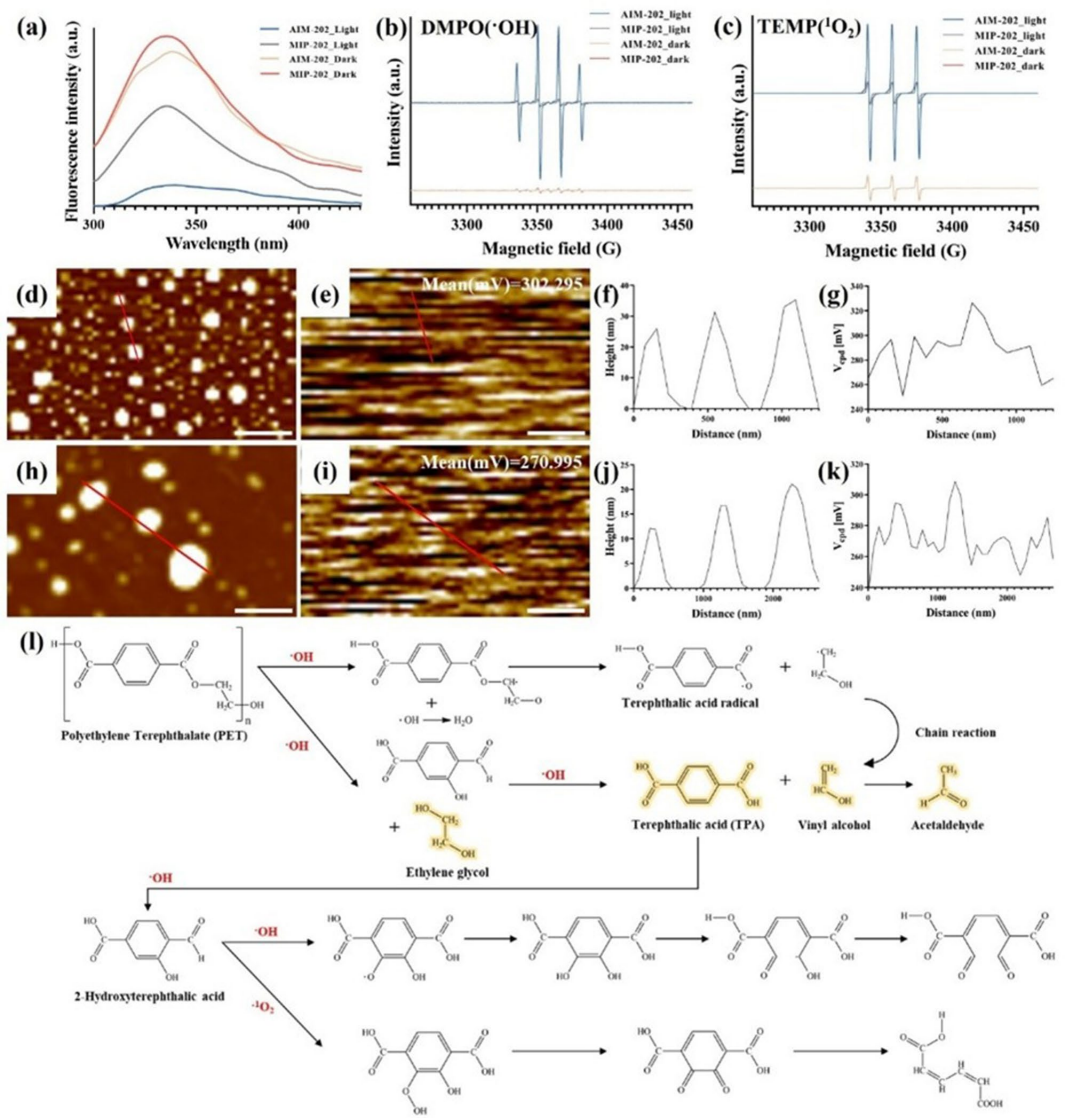
Photophysical and ROS analyses of MIP-202 and AIM-202. (**a**) Baseline corrected PL emission spectra (blank subtracted; baseline set to zero) under UV and dark conditions. (**b**) EPR spectra for hydroxyl radicals (·OH) captured using DMPO. (**c**) EPR spectra for singlet oxygen (^1^O_2_) captured using TEMP. (**d**,** e**) AFM and KPFM images of MIP-202; (**f**,** g**) corresponding height and surface-potential profiles. Scale bars: 1 μm. (**h**,** i**) AFM and KPFM images of AIM-202; (**j**,** k**) corresponding height and surface-potential profiles. Scale bars: 1 μm. (**l**) Schematic of PET degradation mechanisms induced by different ROS

Surface-anchored Ag was introduced to bias interfacial charge flow, while preserving the Zr-MOF lattice. Consistent with this design, AIM-202 exhibited quenched radiative recombination and enhanced electron accumulation on the surface in relation to MIP-202. The steady-state photoluminescence (PL) was lower for AIM-202 under illumination (Fig. 3(a)), indicating more efficient nonradiative charge separation. The lower potential is consistent with the Ag-induced downward band bending at the outer shell, which promotes the accumulation of electrons and their spatial separation from holes in the interior—necessary conditions for interfacial redox [47]. These advantageous electronic properties translate directly to stronger production of ROS. In electron paramagnetic resonance (EPR) spin-trapping experiments, AIM-202 under illumination exhibited substantially stronger signals of hydroxyl radicals with DMPO and ^1^O_2_ with TEMP, while dark signals remained minimal (Fig. 3(b, c)). The Ag ensemble with its Schottky-type Ag⁰ domains and coordinated Ag^δ+^–O sites provide dual electron-capture pathways that cooperatively suppress recombination and increase interfacial electron flux to dissolved O_2_, yielding •O_2_˗ and generating •OH and ^1^O_2_ through downstream reactions [48]. Kelvin probe force microscopy (KPFM) allowed further visualization of this electronic landscape: relative to MIP-202 (mean value ≈ 302 mV), AIM-202 exhibited a reduced and more uniform surface potential (mean value ≈ 271 mV), with smoother profiles across crystallites (Fig. 3(d–k)). Beyond single-probe inferences in previous MOF studies, we link •OH/^1^O_2_ (EPR) to PL quenching and KPFM shifts. This correlation links the Ag0 and Ag^δ+^ surface coordination to charge separation and higher ROS productivity (PL, KPFM, and EPR). The reactions involved in PET oxidation are summarized in Fig. 3(l). Electrophilic •OH drives ester-bond cleavage and ring hydroxylation (producing 2-HTPA, for example), while ^1^O_2_ supports parallel oxidative routes [49, 50]. Chain scission releases products with low molar masses such as terephthalic acid (TPA) and ethylene glycol (EG), with vinyl alcohol/acetaldehyde from secondary reactions. Importantly, this interfacial design can be readily transferred to other water-stable carboxylate MOFs for microplastic photo-oxidation and related ROS-driven processes.

### Optimization of AIM-202 photocatalytic conditions and time-resolved PET degradation analysis

First, we systematically varied the amount of added AIM-202 and examined the chemical fingerprints of PET degradation after 2 weeks. Using the 1410 cm^−1^ aromatic C–H band as reference, four FT-IR ratios tracked oxidation and chain-scission: I(1712/1410) indicates the build-up of carbonyl products from ester cleavage, I(1241/1410) and I(1097/1410) track the growth of C–O and C–O–C from backbone fragmentation, and I(725/1410) reflects perturbation and increased exposure of the phenylene ring. Band assignments and interpretations are summarized in Table S3.

**Fig. 4:**
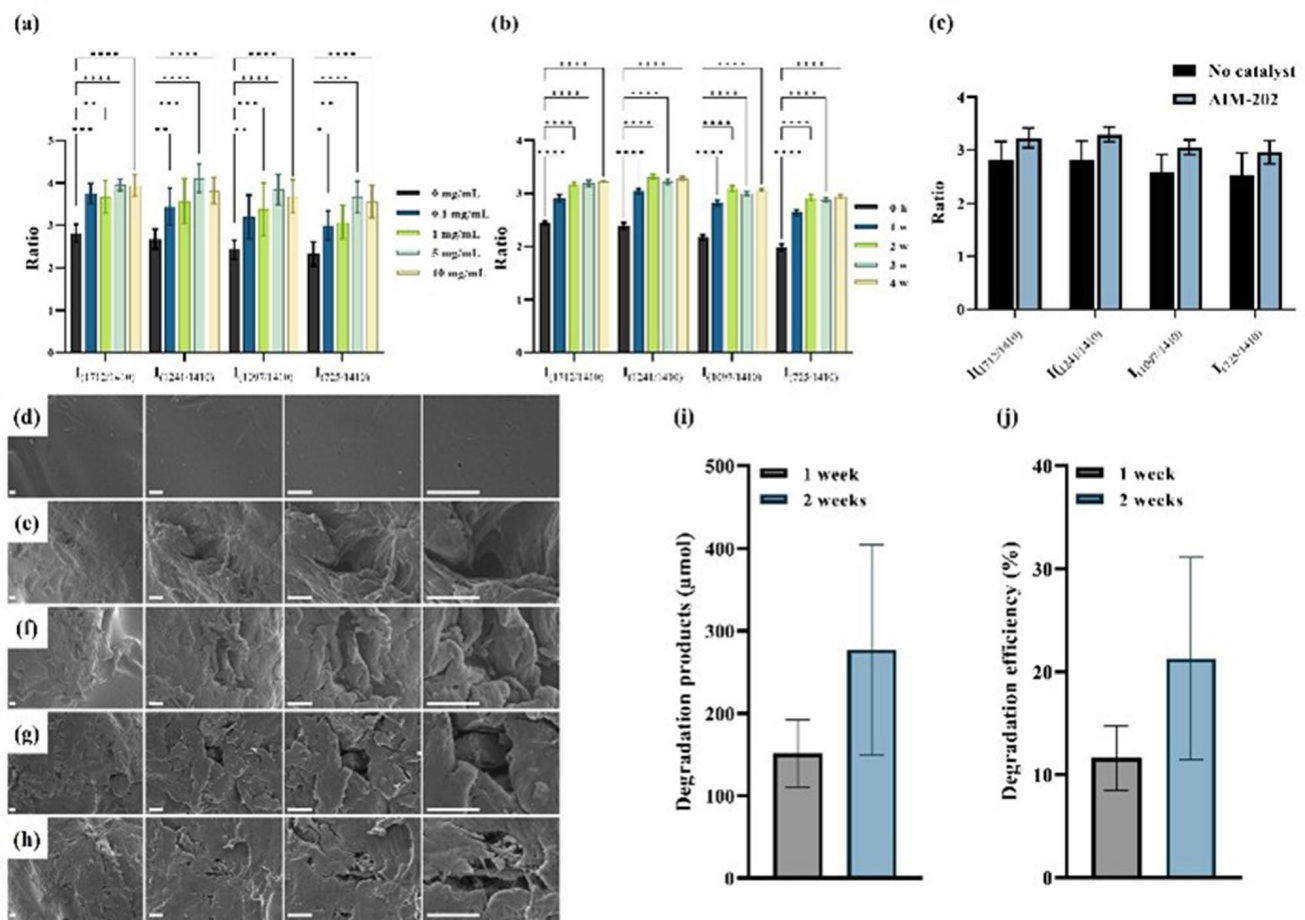
Concentration- and time-dependent performance of AIM-202-mediated PET photodegradation. (**a**) FT-IR intensity ratios I(1712/1410), I(1241/1410), I(1097/1410), and I(725/1410) after Xe-lamp irradiation at different AIM-202 concentrations. (**b**) Time-dependent evolution of the same FT-IR ratios during irradiation. (**c**) FT-IR spectra after 2 weeks for untreated PET, blank (no catalyst + light), and AIM-202 + light (5 mg mL^−1^). (**d–h**) SEM images after 2 weeks for the corresponding conditions, showing pronounced surface erosion only for AIM-202 + light. Scale bars: 5 μm. (**i**) Extracted degradation products (µmol) after 1 and 2 weeks. (**j**) Degradation efficiency (%) based on PET conversion to measurable byproducts

All ratios increased with AIM-202 loading (0.1–5 mg mL^−1^) and plateaued near 10 mg mL^−1^ (Fig. 4(a)); 5 mg mL^−1^ was selected for subsequent tests. The same four FT-IR ratios were used to track bond scission and oxidation over time. After week 1, all four ratios were elevated relative to untreated PET. Using the existing time series in Fig. 4(b), we additionally report apparent pseudo first order kinetics based on FT IR indices, yielding k_app values of 1.23, 0.83, 0.88, and 1.04 week˗1 for I(1712/1410), I(1241/1410), I(1097/1410), and I(725/1410), respectively. Higher time resolution spectra (0 h, 6 h, 12 h, 24 h, 1 week, 2 weeks) are provided in Fig. S6.

ROS flux peaked around week 2, after which degradation slowed but continued until week 4.

Operationally, the inflection around week 2 offers a practical readout that maximizes the signal while conserving time and catalyst. We therefore adopt 2 weeks as the standard endpoint. Expanded, time-resolved ratio plots across all loadings are in Fig. S5, with the underlying spectra in Fig. S6.

To determine whether AIM-202-mediated PET photodegradation generates structural damage and low-molecular-weight products, we combined SEM, 1 H nuclear magnetic resonance (1 H NMR) spectroscopy, and kinetic modeling at a fixed catalyst loading of 5 mg mL^−1^ after irradiation under a Xe lamp for various times (Fig. 4(c–i)). SEM revealed fissures on AIM-202-treated PET surfaces, absent in untreated PET (Fig. 4(c–g)). To quantify the soluble degradation products, 1 H NMR analysis was performed. Integration of three aromatic regions (6.90–9.60 ppm) captured the TPA derivatives and oxidized aromatic species generated by PET backbone cleavage, enabling calculation of the total aromatic byproduct pool (Fig. 4(h)). Representative spectra at weeks 1 and 2, with expanded aromatic windows and peak annotations for TPA/2-HTPA, are in Figs S7 and S8.

After 1 week of irradiation, the overall concentration of measured aromatic and aliphatic compounds was ∼151.40 µmol. This value increased to 277.27 µmol after week 2. The conversion efficiencies were 11.63% at week 1 and 28.68% at week 2 (Fig. 4(i)), indicating steady, quantifiable conversion of PET to soluble aromatic byproducts; these values quantify depolymerization into measured soluble products and do not represent complete mineralization, which would require TOC or CO2 based carbon mass balance.

The linking structure, mechanism, and yield discussed above support a two-stage kinetic model. In the first week involved ROS-induced ester cleavage; week 2 accelerated diffusion and product formation, after which rates tapered due to product layer buildup. Overall, these observations confirm that the coordination-engineered surface electronic properties of AIM-202 drive the deep depolymerization of PET in a chemically verifiable manner under benign conditions. We also verified the structural stability and environmental safety of AIM-202. ICP-OES analysis of the supernatant after 2 weeks showed negligible Ag leaching (Fig. S9). Post-catalysis characterization using PXRD, XPS, and SEM (Fig. S10) confirmed that the crystallinity, oxidation states, and morphology of AIM-202 were preserved, supporting its robustness and reusability.

### PET-derived 2-HTPA as fluorescent probe for Cu^2+^ and Fe^3+^ sensing

Harnessing the chemistry of PET photo-oxidation, we repurposed 2-HTPA as an in situ fluorescent probe for heavy metal monitoring. In this context, “PET-derived” simply indicates that 2-HTPA is a direct product of PET degradation. This molecule is generated by •OH attack on terephthalate linkages and exhibits green emission from its π–π* singlet state. The catechol-like dihydroxy motif of 2-HTPA chelates transition metals and aligns its excited-state energy with low-lying 3d acceptor levels of Cu^2+^ and Fe^3+^, enabling photoinduced electron transfer. The resulting fluorescence quenching enables the sensing of Cu^2+^ and Fe^3+^ without reporters.

**Fig. 5:**
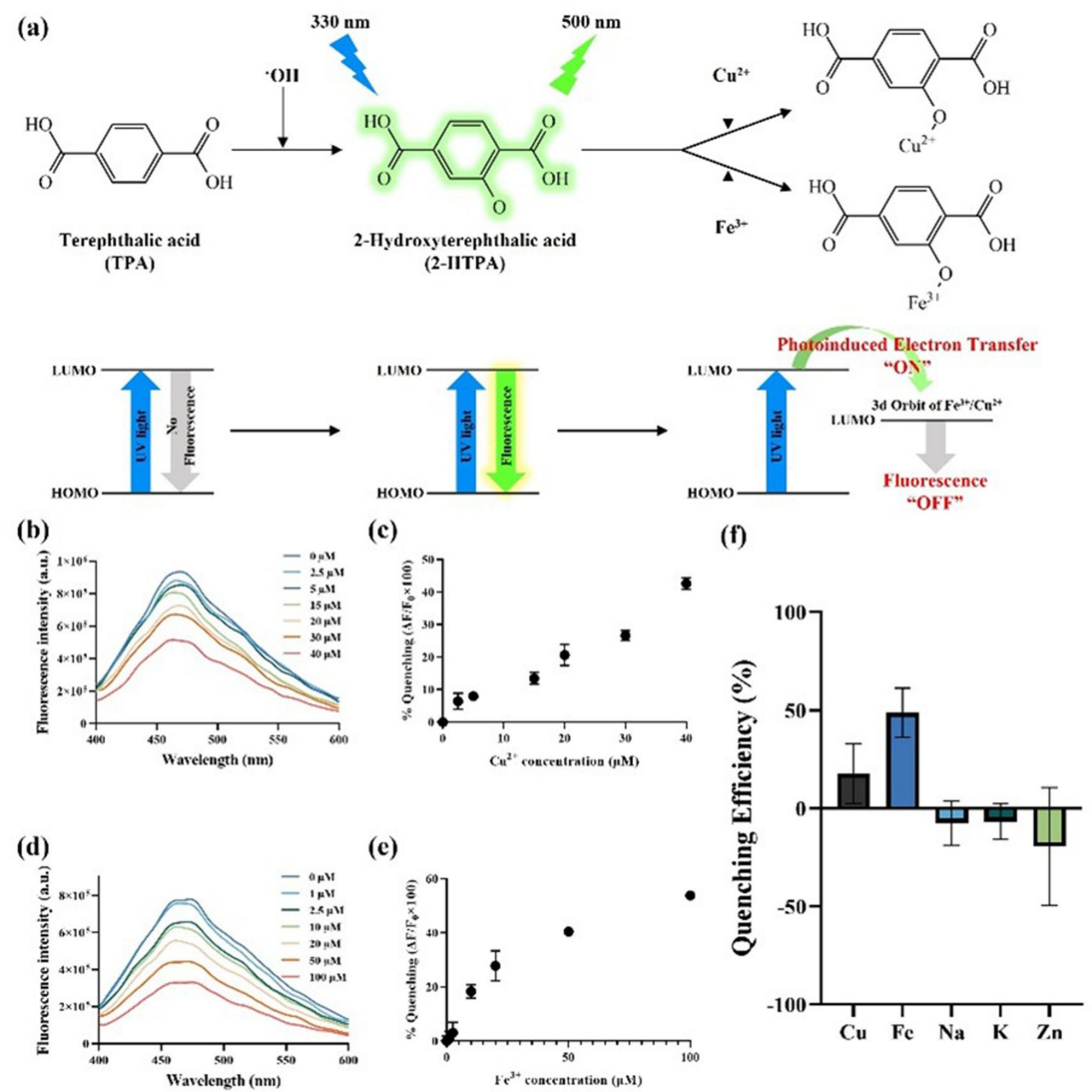
PET-derived fluorescent probe for Cu^2+^ and Fe^3+^ detection. (**a**) Reaction scheme for the conversion of TPA to 2-HTPA and the photoinduced electron-transfer quenching pathway with Cu^2+^ and Fe^3+^. (**b**) Fluorescence spectra of 2-HTPA with increasing Cu^2+^ concentration, showing monotonic intensity loss near 500 nm. (**c**) Quenching efficiency (ΔF/F₀ × 100) versus Cu^2+^ concentration, displaying an increasing trend that includes an approximately linear regime at low concentrations. (**d**) Fluorescence spectra of 2-HTPA with increasing Fe^3+^ concentration, showing stronger quenching per µM than Cu^2+^. (**e**) Quenching efficiency (ΔF/F₀ × 100) versus Fe^3+^ concentration, showing a steep initial slope and a broader dynamic range. (**f**). Selectivity of PET derived 2 HTPA toward Cu^2+^ and Fe^3+^ in the presence of interfering ions. Quenching efficiency (ΔF/F0 × 100) of PET derived 2 HTPA upon exposure to Cu^2+^ Fe^3+^ and background ions (Na + K+ Zn2+) at a final concentration of 100 µM after 10 min incubation at 25 °C (Ex 370 nm, Em 450 nm)

With an increase in the Cu^2+^ concentration, the emission of 2-HTPA near 500 nm decreased monotonically with negligible band shift, consistent with PET-driven quenching (Fig. 5(b)). Compared to Cu^2+^, Fe^3+^ produced a stronger quenching response over a broader dynamic range (Fig. 5(d)). The quenching efficiencies (ΔF/F₀ × 100) increased with the metal concentration and were approximately linear at low concentrations, with Fe^3+^ exhibiting a steeper initial slope than Cu^2+^ (Fig. 5(c, e)). The curvature at higher concentrations suggested a combination of dynamic and static contributions. Because 2-HTPA is produced in PET upcycling, the sensing signal scales with the ROS flux that drives depolymerization, directly coupling environmental remediation with contaminant monitoring.

In this platform, the selectivity in heavy metal detection is rooted in ligand field preferences of the o-dihydroxybenzene unit, which favors hard, high-valent centers such as Fe^3+^ and to a lesser extent Cu^2+^ under near-neutral conditions.

**Fig. 6:**
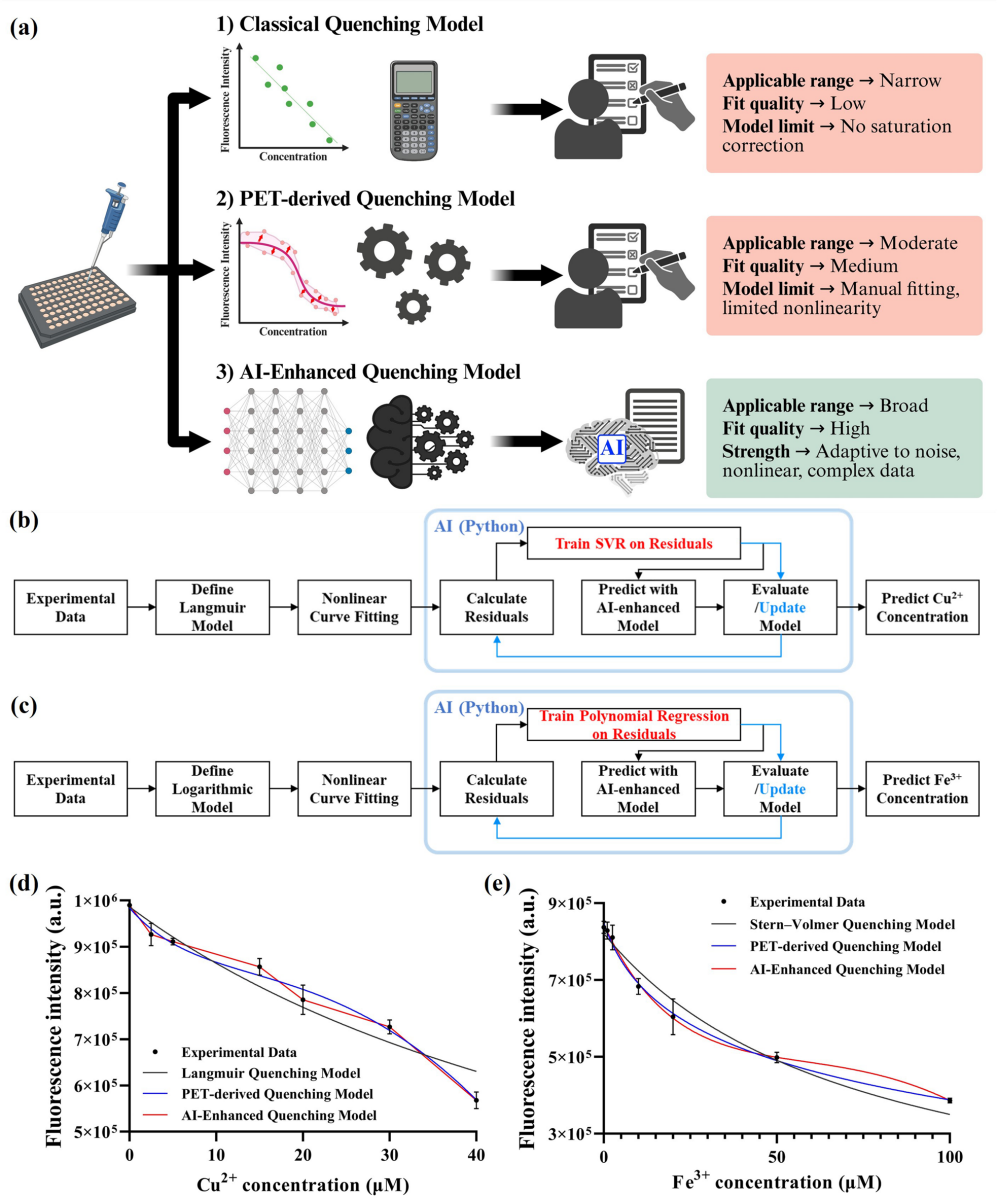
AI-enhanced modeling for fluorescence-based quantification of Cu^2+^ and Fe^3+^ using PET-derived 2-HTPA. Training and test performance (LOOCV) is summarized in Table S6. (**a**) Comparison of classical, PET-derived, and AI-enhanced models with respect to range, fit quality, and adaptability. (**b**,** c**) Schematic workflows for AI-assisted model development, using residual correction with SVR for Cu^2+^ and polynomial regression for Fe^3+^. (**d**,** e**) Comparison of classical, PET-derived, and AI-enhanced quenching models against experimental fluorescence data for (**d**) Cu^2+^ and (e) Fe^3+^

### Spectral analysis and quantitative modeling for Cu^2+^ detection

2-HTPA exhibits intense emission near 450 nm upon ultraviolet excitation. Its fluorescence is specifically quenched by Cu^2+^. To evaluate this quenching behavior, fluorescence spectra were recorded while increasing the Cu^2+^ concentrations. The emission decreased with the increase in concentration, with the strongest attenuation near the ~ 450 nm maximum, confirming a specific interaction (Fig. 5(b)).

The quenching rate (quantified as ΔF∕F₀ × 100, Fig. 5(c)) increased at low Cu^2+^ concentrations and approached saturation at higher levels, exhibiting a nonlinear trend.

#### Langmuir quenching model

This model assumes static quenching:

1 $$\:\begin{array}{c}F\left(Q\right)=\frac{{F}_{0}}{\left(1+K\cdot\:Q\right)}\end{array}$$

Here, F(Q) is the fluorescence intensity at quencher concentration Q (µM), F₀ is the fluorescence intensity at Q = 0, and K (µM^˗1^) is the Langmuir binding constant. Nonlinear curve fitting using this model yielded 𝐹_0_ = 986,157 and 𝐾 = 0.01411, with a coefficient of determination (𝑅^2^) of 0.9354 and a root-mean-square error (RMSE) of 33,739.08.

#### PET-derived langmuir-quadratic quenching model

To better capture the observed saturation behavior, we propose a Langmuir-quadratic hybrid model incorporating a second-order element into the standard Langmuir equation. This model is expressed as follows:

2 $$\:\begin{array}{c}F\left(Q\right)=\frac{{F}_{0}}{\left(1+K\cdot\:Q\right)}+a{\cdot\:Q}^{2}+b\cdot\:{Q}^{2.}\end{array}$$

Curve fitting produced the following parameters: 𝐹_0_ = 984,146, 𝐾 = 0.04701, 𝑎 = −467.0, and 𝑏 = 243. This model attained an 𝑅^2^ of 0.9909 and an RMSE of 12649.73, indicating enhanced prediction accuracy.

#### Artificial intelligence assisted Langmuir-quadratic SVR quenching model (Machine learning assisted regression)

To address the constraints of traditional physicochemical models and rectify the experimental residuals, a hybrid model integrating AI-driven support vector regression (SVR) was developed. This model is based on the Langmuir-quadratic model but employs AI to minimize the residuals. It is expressed as follows:

3 $$\:\begin{array}{c}{F}_{final}\left(Q\right)={F}_{base}\left(Q\right)+SVR\left(Q\right).\end{array}$$

Here, 𝐹base(𝑄) denotes the fluorescence intensity predicted by the Langmuir-quadratic model, whereas SVR(𝑄) serves as a correction function derived from the residuals between the anticipated and experimental values. Residuals were modeled ε-insensitive SVR so that

3 $$\:\begin{array}{c}SVR\left(Q\right)={\sum\:}_{i=1}^{{N}_{SV}}{\alpha\:}_{i}k\left(Q,\:{Q}_{i}\right)+b,\end{array}$$

where 𝑄_i_ represents the support vectors, and k is the kernel function. This correction term was executed using the scikit-learn module in Python. The final model demonstrated exceptional agreement to experimental data, with 𝑅^2^ = 0.9999 and RMSE = 94.01 (Fig. 6(d)). Model definitions and fit metrics for Cu^2+^ are summarized in Table S4.

This model accurately addresses the nonlinear saturation behavior at high concentrations. Thus, it can effectively surmount the interpretative constraints of Langmuir-based models.

### Spectral analysis and quantitative modeling for Fe^3+^ detection

Although 2-HTPA demonstrates sensitivity to Cu^2+^, significant fluorescence quenching can also be induced by Fe^3+^ ions. We obtained the fluorescence spectra of 2-HTPA at different Fe^3+^ concentrations. There was a swift reduction in fluorescence intensity even at minimal concentrations, signifying high quenching efficiency.

We calculated the fluorescence quenching rate in the same way as for Cu^2+^ analysis (ΔF/F₀ × 100); the results are plotted in Fig. 5(e). The quenching rate escalated significantly with increasing Fe^3+^ concentration, exhibiting an exponential rather than a linear trend. The model definitions and fit metrics for Fe^3+^ are presented in Table S5.

#### Stern–Volmer quenching model

The classical Stern–Volmer model is expressed as

5 $$\:\begin{array}{c}\frac{{F}_{0}}{F}=1+{K}_{SV}\left[Q\right],\:\end{array}$$

where 𝐹0, 𝐹, 𝑄 and 𝐾SV denote standard Stern-Volmer parameters. When the Fe^3+^ concentration surpassed 10 µM, the model overestimated the fluorescence (Fig. 6(e)).

#### PET-derived logarithmic quenching model

To accurately characterize the observed nonlinearity and saturation behavior, we propose the following innovative PET-derived logarithmic quenching model:

6 $$\:\begin{array}{c}F\left(Q\right)={F}_{0}\cdot\:\left(1-\alpha\:\cdot\:{{log}}_{e}\left(1+\gamma\:Q\right)\right),\end{array}$$

where 𝛼 is a coefficient signifying the highest quenching efficiency, and 𝛾 is a nonlinear parameter that reflects concentration sensitivity. Nonlinear regression analysis produced the following optimal parameters: F_0_ = 849,326, α = 0.1884, and γ = 0.1688. This model exhibited outstanding fitting ability, with 𝑅2 = 0.9960 and RMSE = 138.8. In Fig. 6(e), predictions from the PET-derived logarithmic quenching model show better correlation with experimental data over the entire concentration range.

#### AI-enhanced polynomial regression quenching model

To maximize prediction accuracy, a high-order polynomial regression model utilizing Python was created for the Fe^3+^ dataset, by adding high-order correction terms to the aforementioned logarithmic model as follows:

7 $$\:\begin{array}{*{20}{c}} \begin{aligned} F\left( Q \right) = & {F_0} \cdot \:\left( {1 - \alpha \: \cdot \:ln\left( {1 + \delta \: \cdot \:Q} \right)} \right) \\ & + a \cdot \:Q + b \cdot \:{Q^2} + c \cdot \:{Q^3}. \\ \end{aligned} \end{array}$$

This model demonstrated exceptional fitting performance, with 𝑅2 = 0.9962 and RMSE = 10,113.63. It exhibited a consistent curvature over the entire concentration range and precisely replicated the nonlinear fluorescence quenching behavior caused by Fe^3+^ ions. The model effectively addresses nonlinear saturation and quenching behaviors at high concentrations—a phenomenon inadequately explained by traditional theoretical models.

These results demonstrate that while the classical Langmuir and Stern–Volmer models provide a baseline description of fluorescence quenching, they fail to capture the nonlinear response at higher quencher concentrations. The PET-derived hybrid models introduce correction terms that significantly increase fitting accuracy while maintaining mechanistic interpretability. Taken together, these findings establish a generalized modeling strategy that is not limited to a single ion but can be readily extended to different quenching systems for environmental-sensing applications. Finally, to highlight the unique position of our work within the field, we benchmarked the performance and sensing integration of AIM-202 against representative Ag-based and Zr-based photocatalysts reported in the literature (Table S7). Unlike systems limited to high-temperature degradation or lacking sensing capabilities, AIM-202 offers a distinct dual-functionality under mild conditions.

## Conclusions

This study established a waste-to-sensor paradigm in which microplastic upcycling, photocatalysis, and analytics were designed together around an Ag/Zr-MOF platform. By coordinating Ag at surface carboxylate/O sites while preserving the Zr-aspartate lattice, AIM-202 retains the framework stability but possesses engineered surface electronic properties that favor interfacial charge separation and O_2_ activation. This is proven by converging spectroscopic and structural fingerprints, and the electronic consequences are revealed by PL quenching, KPFM band bending, and EPR signatures of •OH and ^1^O_2_. Owing to these favorable electronic properties, AIM-202 at the optimal loading of 5 mg mL^−1^ achieved PET depolymerization, corroborated by increased FT-IR intensity ratios, progressive surface erosion and porosity, quantifiable soluble products, and conversion efficiencies of ~ 11.6% and ~ 28.7%, respectively. Collectively, these results define the core contribution: a lattice-preserving, surface-engineered Ag/Zr-MOF that couples verifiable electronic-structure changes with ROS-driven PET depolymerization.

Leveraging the degradation chemistry, we repurposed 2-HTPA (a fluorophore from PET degradation) as an intrinsic reporter for heavy metal monitoring, because its emission is quenched by Cu^2+^ and Fe^3+^ via chelation-assisted photoinduced electron transfer. The resulting nonlinear concentration-response curves were fitted to physics-guided models and refined by lightweight machine-learning correctors. For Cu^2+^, a Langmuir-quadratic base model augmented with SVR residual correction achieved near-perfect agreement. For Fe^3+^, a logarithmic PET-derived model achieved further improvement using high-order correction terms. These hybrid models retain the mechanistic parameters while expanding the range and accuracy, enabling robust back-calculation from single-point intensities and deployment on portable readers or smartphones.

More broadly, our results imply that surface coordination engineering can tune water-stable, carboxylate-rich MOFs, delivering a high ROS flux with minimal noble-metal loading while preserving porosity and crystallinity of the parent MOF. The result is a closed-loop platform in which the ROS chemistry that degrades microplastics also generates molecular probes to monitor co-contaminants in situ. Practical deployment will use immobilized AIM-202 and periodic model retraining to manage durability, trace Ag release, and matrix effects. To achieve this, further studies are needed regarding the durability in complex matrices, long-term Ag retention, solar spectrum harvesting in flow photoreactors, and multiplex quantification in the presence of interferents. With incremental data-driven retraining of hybrid models and modular surface chemistry to host alternative co-catalysts or recognition motifs, the waste-to-sensor strategy outlined herein provides a scalable route for integrated environmental remediation and analytics.

## Electronic Supplementary Material

Below is the link to the electronic supplementary material.

Supplementary Material 1

Supplementary Material 2

Supplementary Material 3

## Data Availability

All data generated or analyzed during this study are included in this published article and its supplementary information files.
