# Supplementary material for: Waste-to-sensor upcycling of polyethylene terephthalate over Ag/Zr-MOF photocatalyst for microplastic degradation and AI-assisted heavy metal detection
Source: J Biol Eng. 2026 Apr 25;20:100. doi: 10.1186/s13036-026-00683-4 (PMC13248307; doi:10.1186/s13036-026-00683-4)
Supplement: Supplementary file 1 — Supplementary Material 1 [file 13036_2026_683_MOESM1_ESM.docx]

Supporting Information

Waste-to-Sensor Upcycling of Polyethylene Terephthalate over Ag/Zr-MOF Photocatalyst for Microplastic Degradation and AI-Assisted Heavy Metal Detection

Minse Kim, Kisung Lee, Jaewon Lee, Jeong-Ann Park, Kwang Suk Lim, Suk-Jin Ha and Hyun-Ouk Kim*

Table S1. Elemental compositions of MIP-202 and AIM-202 determined via SEM–EDS. Weight percentages (wt%) of C, N, O, Zr, and Ag in MIP-202 and AIM-202, as measured via EDS.

| **Element** | **MIP-202** | | **AIM-202** | |
| --- | --- | --- | --- | --- |
|  | **Wt (%)** | **At (%)** | **Wt (%)** | **At (%)** |
| **C** | **18.05** | **40.30** | **9.80** | **25.85** |
| **N** | **4.22** | **8.09** | **4.21** | **9.52** |
| **O** | **20.82** | **34.89** | **22.27** | **44.10** |
| **Zr** | **56.90** | **16.72** | **33.87** | **11.76** |
| **Ag** | **0.00** | **0.00** | **29.84** | **8.77** |
| **Total** | **100.00** | **100.00** | **100.00** | **100.00** |


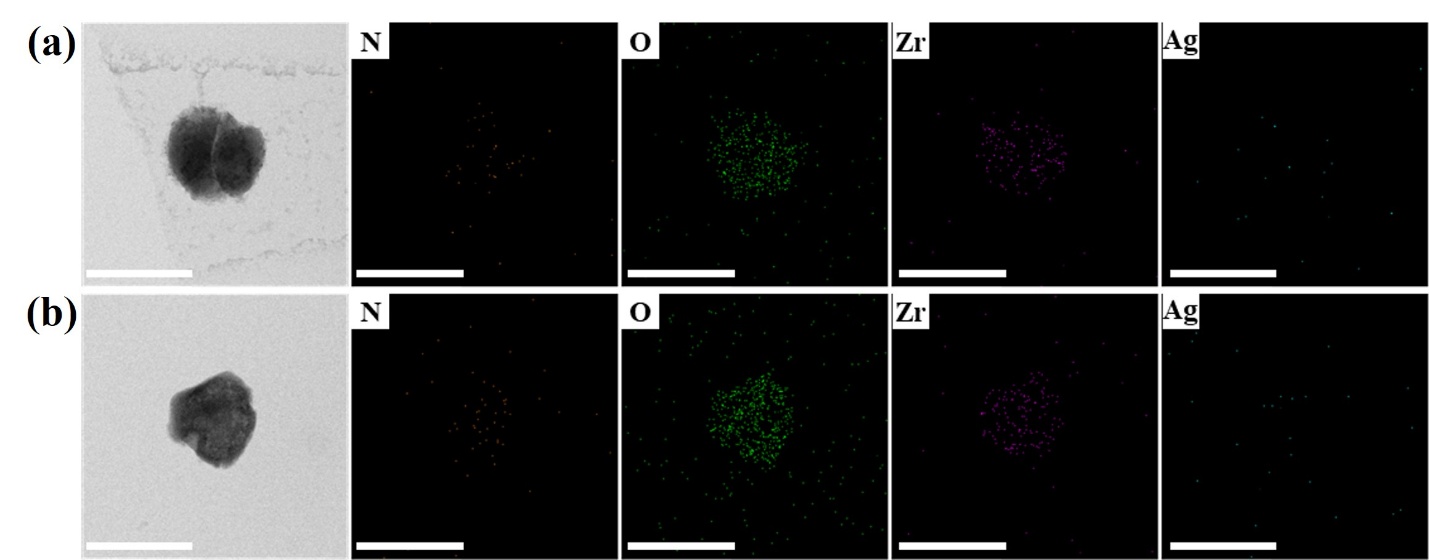


Figure S1. TEM–EDS analysis of MIP-202 and AIM-202. (a, b) TEM images and EDS elemental maps of (a) MIP-202 and (b) AIM-202, confirming the presence of N (orange), O (green), and Zr (purple) in both samples, with a distinct Ag signal (cyan) observed only in AIM-202. Scale bars: 500 nm.

Table S2. Elemental compositions of MIP-202 and AIM-202 determined via TEM–EDS. Weight percentages (wt%) of N, O, Zr, and Ag in MIP-202 and AIM-202, as measured via EDS.

| **Wt(%)** | **MIP-202** | **AIM-202** |
| --- | --- | --- |
| **N** | **19.5** | **17.8** |
| **O** | **46.4** | **49.9** |
| **Zr** | **33.2** | **29.7** |
| **Ag** | **0.9** | **2.6** |
| **Total** | **100** | **100** |

**
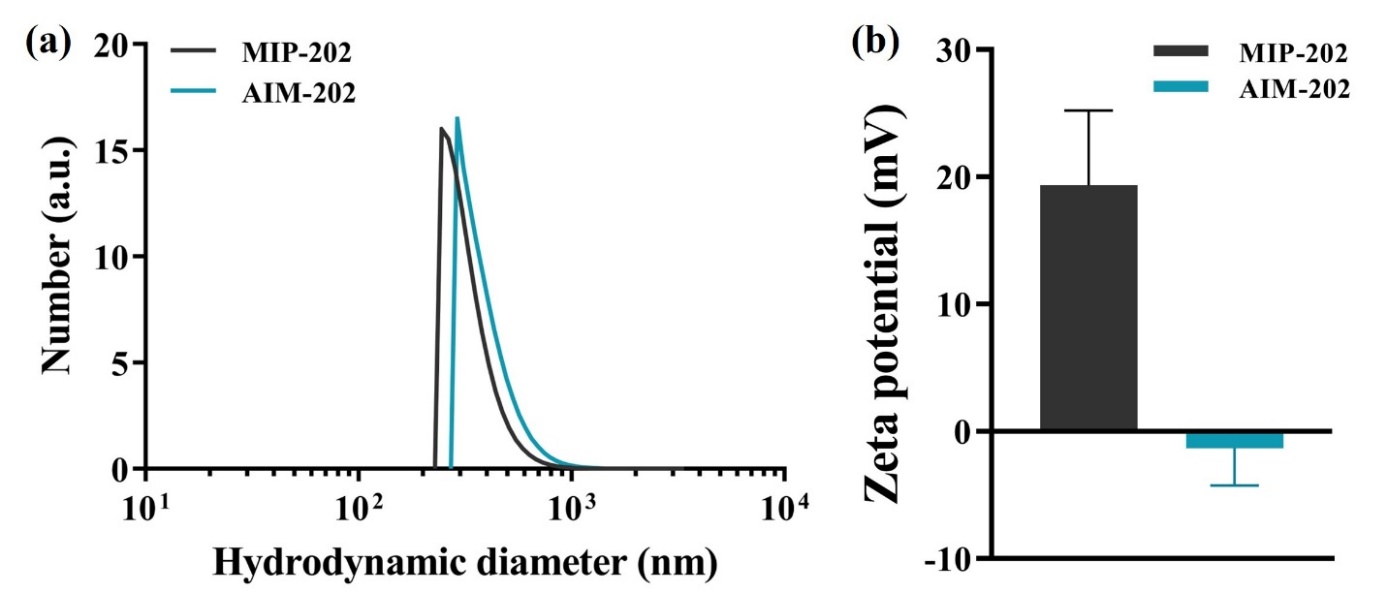
**

Figure S2. Size distribution and surface charge analysis of MIP-202 and AIM-202. (a, b) DLS and zeta-potential analysis of (a) MIP-202 and AIM-202 hydrodynamic diameters and (b) their surface charges, showing size similarity but a charge shift from positive (MIP-202) to near-neutral/negative (AIM-202).

Table S3. Assignment of FT-IR absorption bands and corresponding degradation mechanisms. Summary of characteristic FT-IR bands observed during PET photodegradation and their associated functional-group changes and oxidation pathways.

| **Wavenumber (cm⁻¹)** | **Assigned bond** | **Functional group or mode** | **Functional interpretation related to PET degradation** |
| --- | --- | --- | --- |
| **1712** | C=O stretch | Carbonyl group (ester/acid) | Ester-bond cleavage or oxidation to aldehyde/carboxylic acid |
| **1410** | CH₂ bending | Reference peak (aliphatic C–H) | Stable internal reference for intensity normalization |
| **1241** | C–O stretching | Ester linkage (O–C–O) | Ester-bond cleavage within PET backbone, consistent with formation of TPA |
| **1097** | C–O–C stretch | Asymmetric ether/ester stretch | Accumulation or exposure of ester-containing degradation products such as TPA |
| **725** | C–H bending | Aromatic ring (phenylene unit) | Accumulation or exposure of aromatic degradation products such as TPA |


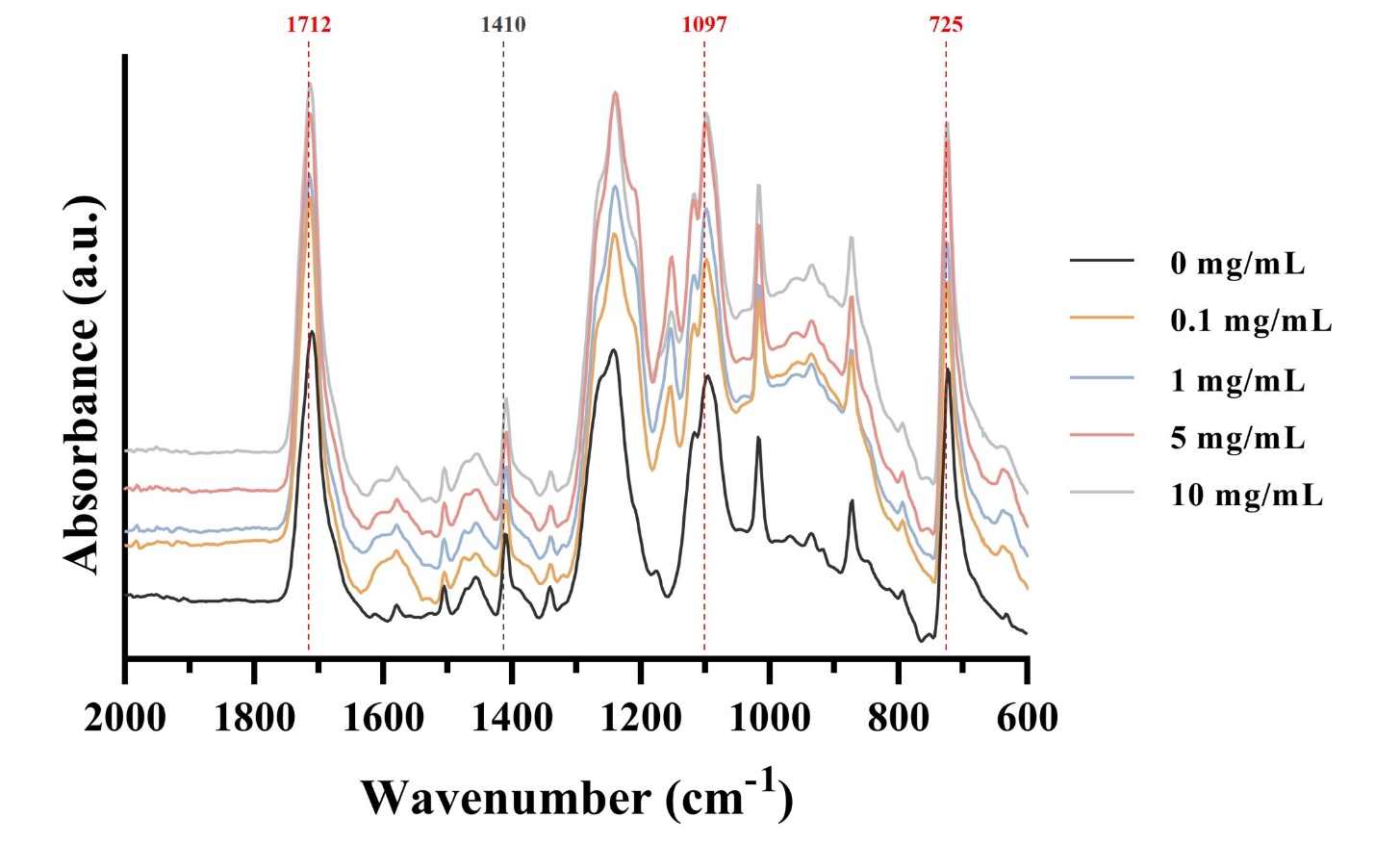


Figure S3. FT-IR analysis of PET degradation by AIM-202 under different concentrations. FT-IR spectra of PET after 2 weeks of light irradiation in the presence of AIM-202 at varying concentrations (0.1, 1, 5, and 10 mg/mL), compared to untreated PET. Characteristic absorption bands at 1712, 1410, 1241, 1097, and 725 cm⁻¹ were tracked to monitor structural changes.


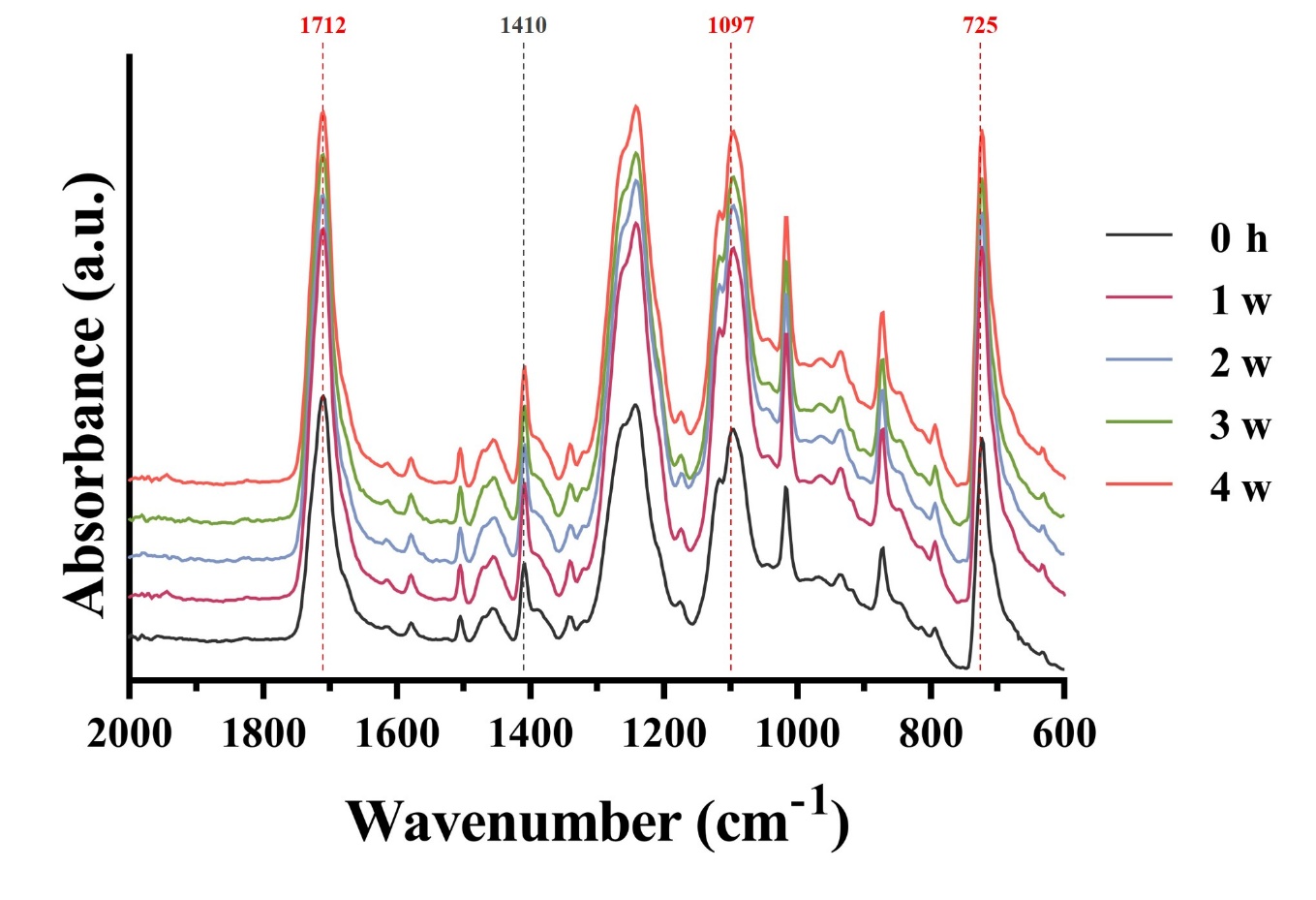


Figure S4. FT-IR analysis of PET degradation by AIM-202 under different irradiation times. Time-dependent FT-IR spectra of PET samples treated with AIM-202 (5 mg/mL) and irradiated under a Xe lamp for different durations (0 h, 1 week, 2 weeks, 3 weeks, and 4 weeks). Absorption bands corresponding to carbonyl (1712 cm⁻¹), reference (1410 cm⁻¹), ether (1097 cm⁻¹), and aromatic C–H (725 cm⁻¹) vibrations were monitored to evaluate the chemical evolution during photocatalytic degradation.


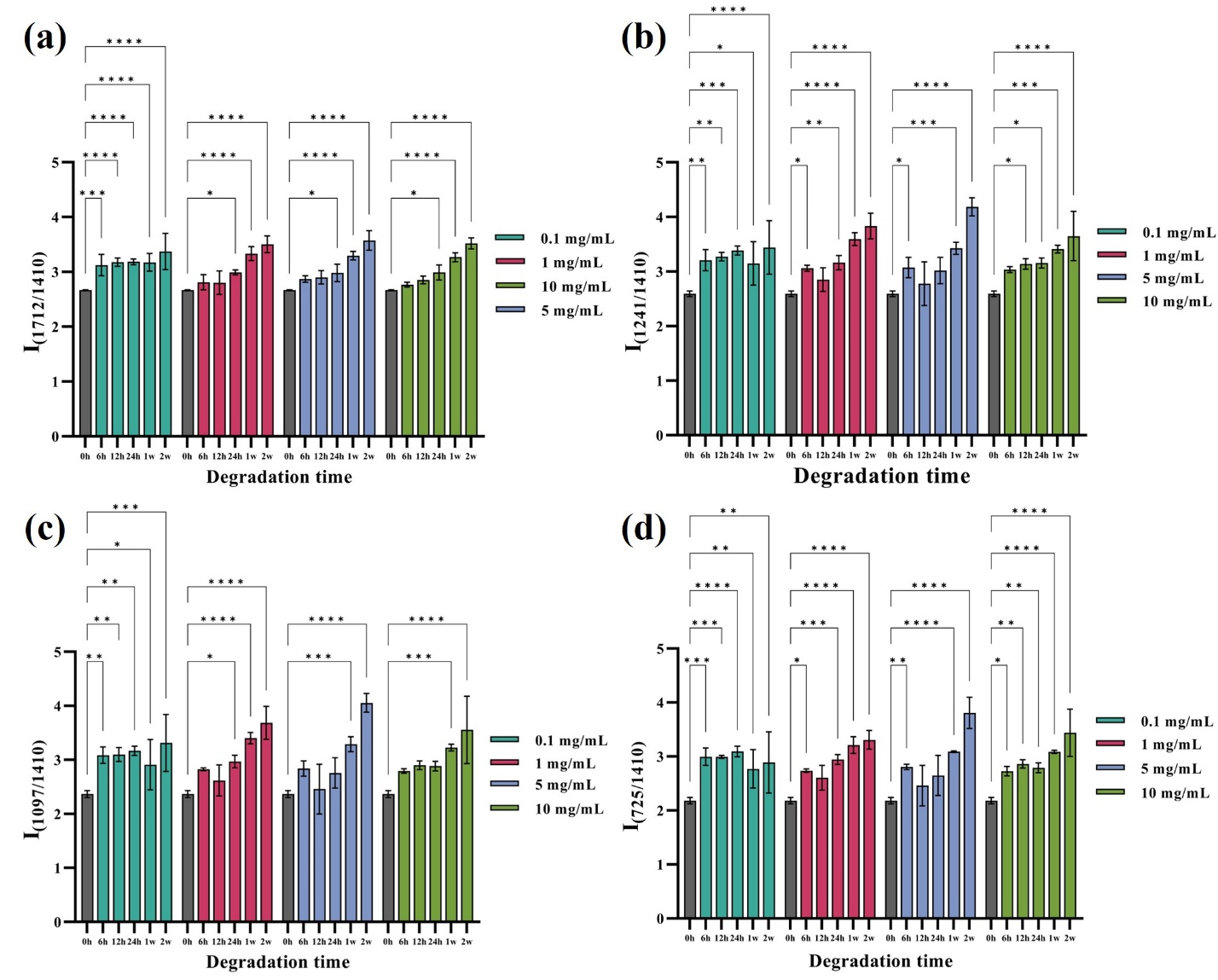


Figure S5. Time-resolved FT-IR intensity ratios of PET degradation at varying AIM-202 concentrations. (a–d) Intensity ratios of PET functional groups after exposure to AIM-202 at different concentrations (0.1–10 mg/mL) under Xe-lamp irradiation for up to 2 weeks. (a) Carbonyl groups (I(1712/1410)), (b) ester groups (I(1241/1410)), (c) ether groups (I(1097/1410)), and (d) aromatic C–H groups (I(725/1410)). All intensities are normalized to the 1410 cm⁻¹ reference band, and statistical significance is indicated as *p < 0.05, **p < 0.01, ***p < 0.001, ****p < 0.0001.


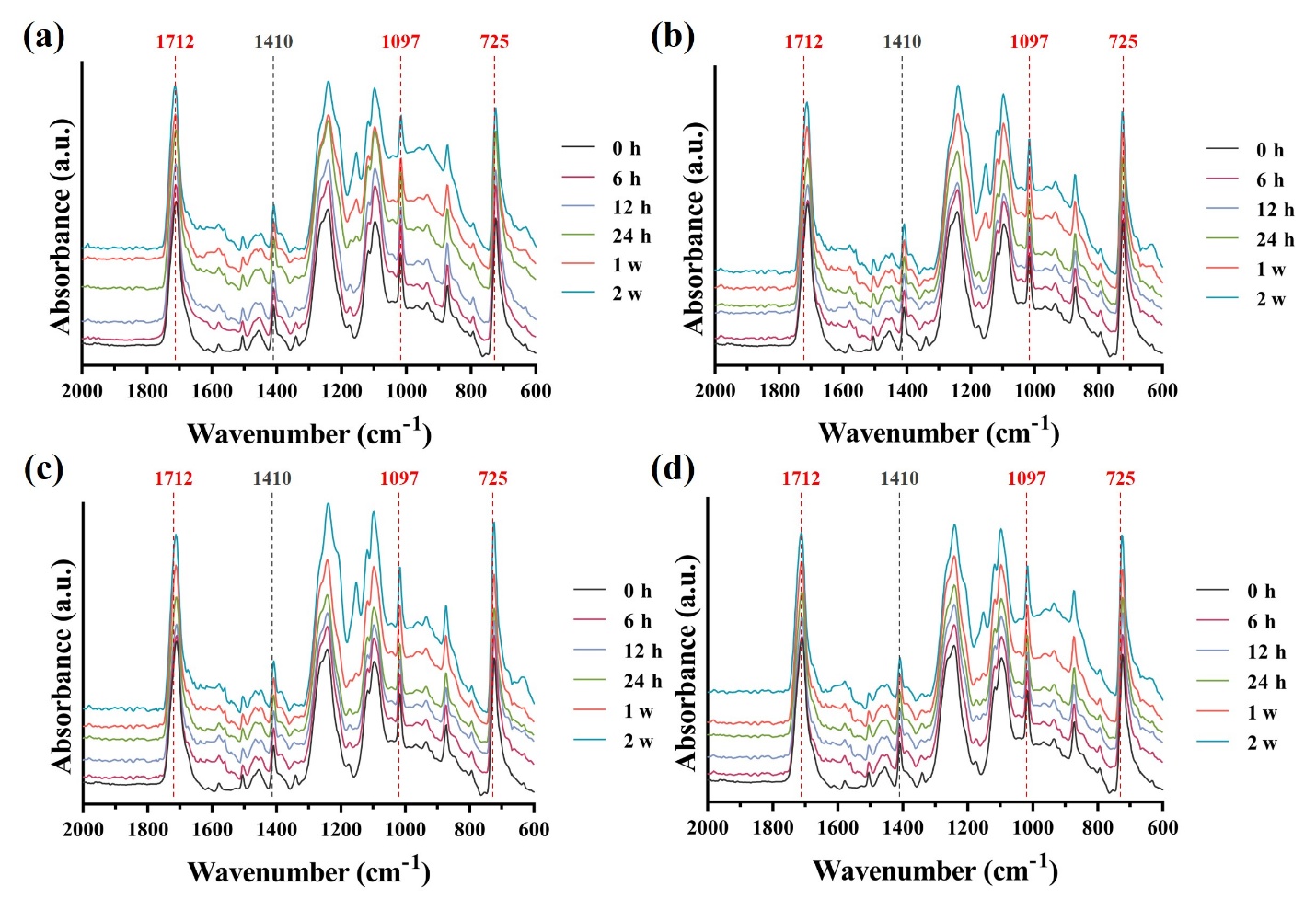


Figure S6. Time-dependent FT-IR spectra of PET degradation by AIM-202 under Xe-lamp irradiation. (a–d) FT-IR spectra of PET treated with AIM-202 at different concentrations and exposed to Xe-lamp irradiation for various time intervals (0 h, 6 h, 12 h, 24 h, 1 week, 2 weeks). (a) 0.1 mg/mL AIM-202, (b) 1 mg/mL AIM-202, (c) 5 mg/mL AIM-202, and (d) 10 mg/mL AIM-202. The evolution of characteristic absorption peaks at 1712 cm⁻¹ (C=O), 1241 cm⁻¹ (C–O), 1097 cm⁻¹ (C–O–C), and 725 cm⁻¹ (aromatic C–H) was tracked to monitor photocatalytic PET degradation.


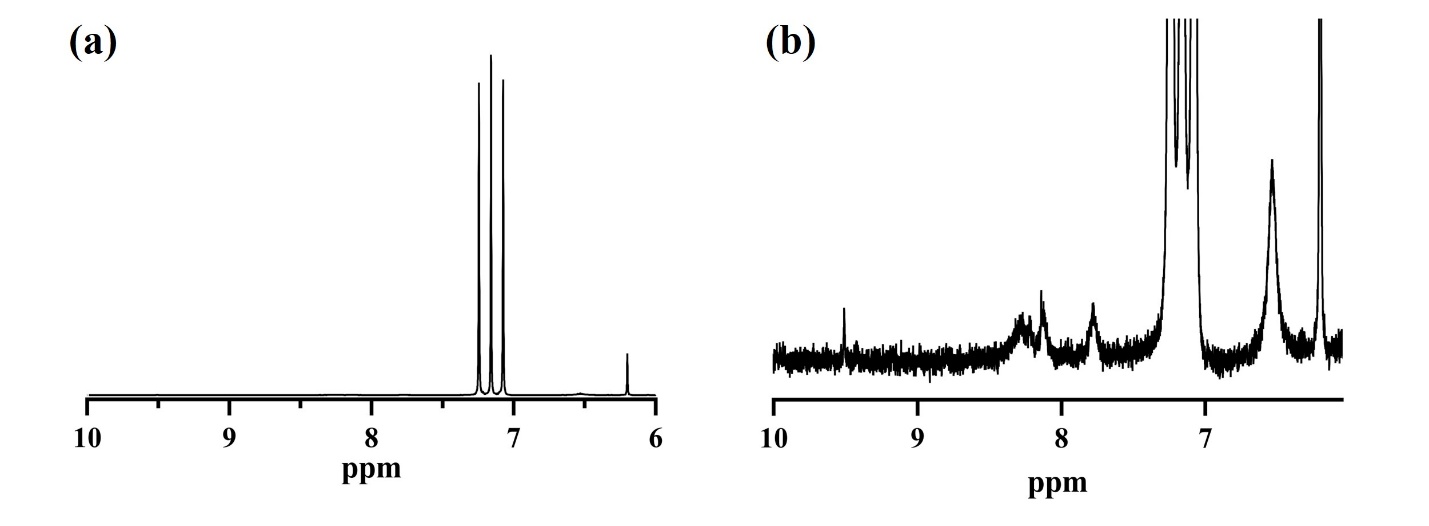


Figure S7. ¹H NMR spectra of PET photodegradation products in d-DMSO. (a–b) ¹H NMR spectra of PET degradation products obtained after AIM-202 (5 mg/mL) treatment under Xe-lamp irradiation. (a) Spectra of degradation products after week 1, (b) expanded aromatic region highlighting characteristic signals corresponding to terephthalic acid (TPA) and 2-hydroxyterephthalic acid (2-HTPA).


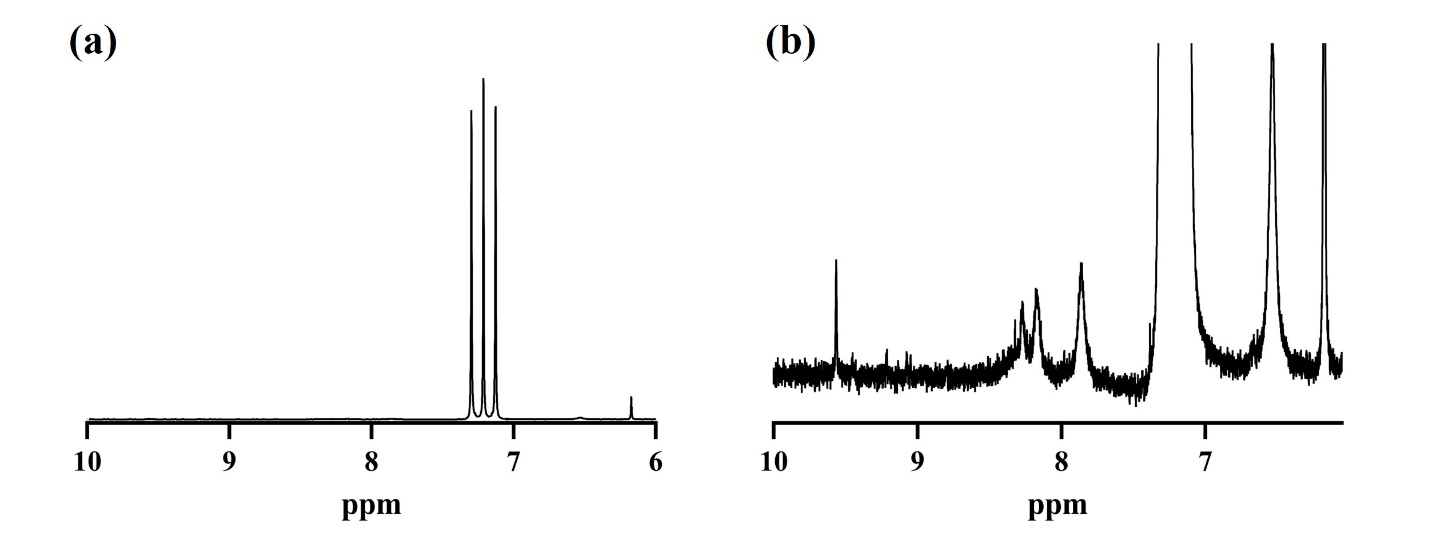


Figure S8. ¹H NMR spectra of PET photodegradation products in d-DMSO. (a–b) ¹H NMR spectra of PET degradation products obtained after AIM-202 (5 mg/mL) treatment under Xe-lamp irradiation. (a) Spectra of degradation products after week 2, and (b) Expanded aromatic region after week 2, showing distinctive signals assigned to TPA and 2-HTPA.

**
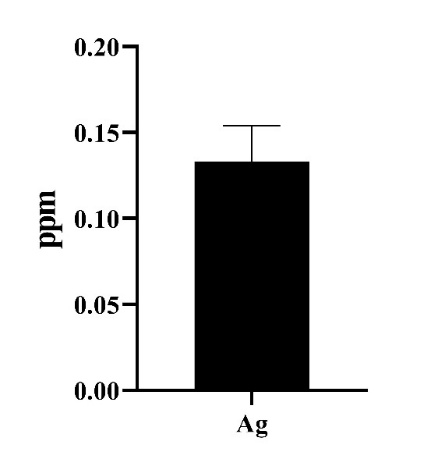
**

**Figure S9. Ag concentration in post-reaction supernatant after photocatalytic PET degradation.** Ag in the supernatant was quantified by ICP-OES after photocatalysis using AIM-202 (5 mg/mL) under light irradiation for 2 weeks. Bars represent mean ± SD (n = 3). A statement on multi-cycle leaching stability is provided in the Discussion as a limitation and future work.


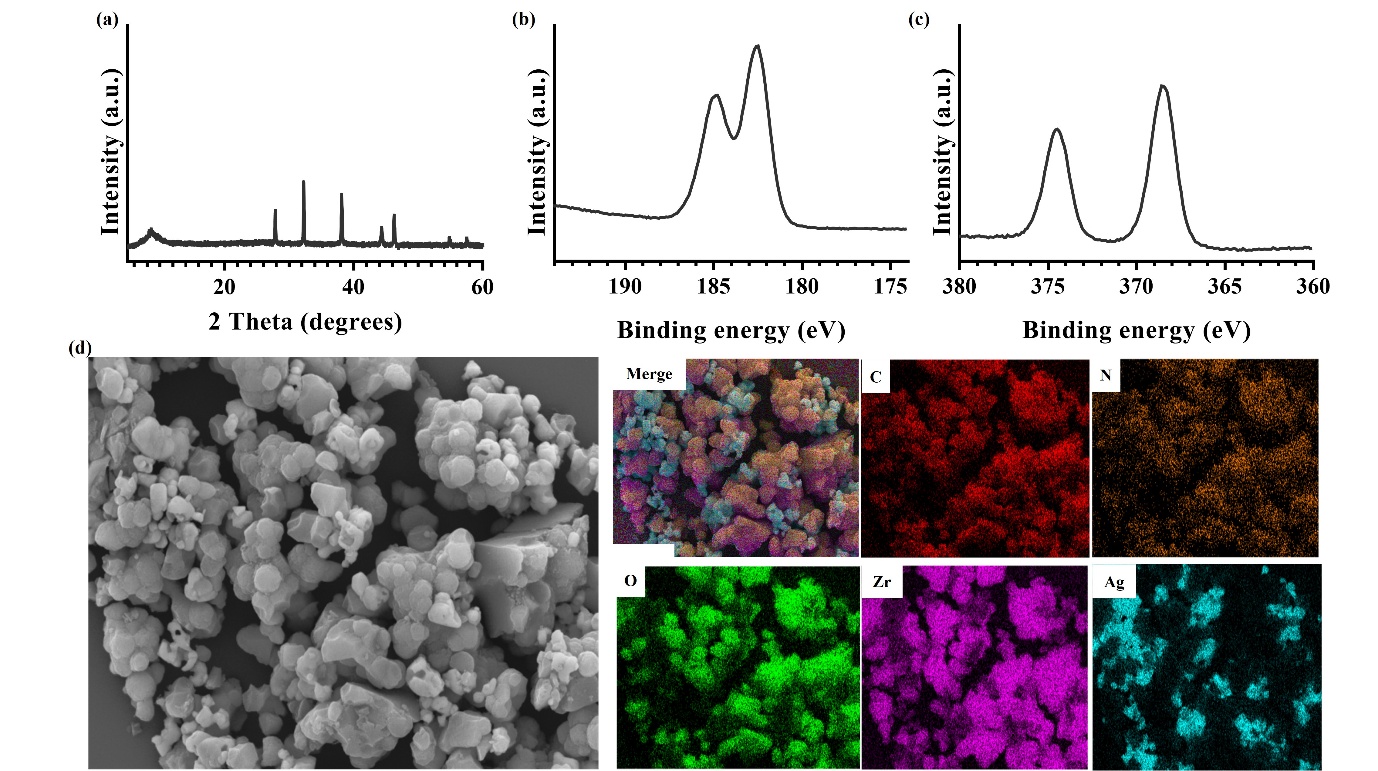


Figure S10. Post photocatalysis stability of AIM-202 after 2 week PET photocatalysis (5 mg/mL): (a) PXRD crystallinity retention, (b) XPS Ag and (c) Zr core levels, and (d) SEM/EDS morphology and elemental distribution.

**
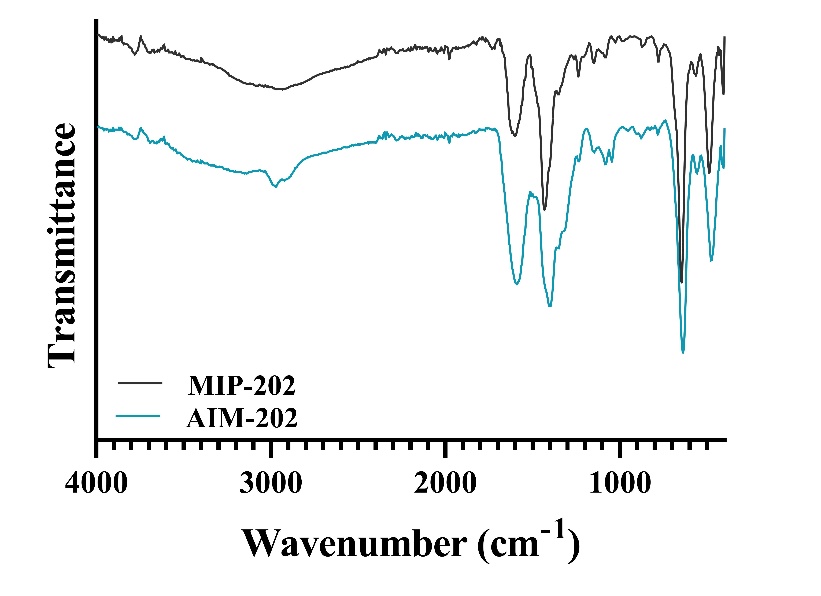
**

**Figure S11.** FTIR spectra of MIP-202 and AIM-202 over 4000 to 400 cm⁻¹.


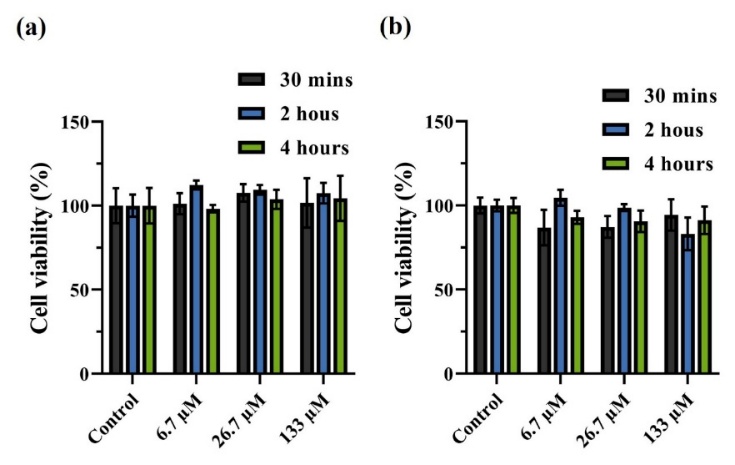


**Figure S12.** In vitro cell viability assessment of MOF. (a) MIP-202 and (b) AIM-202 at different concentrations over various incubation times was evaluated using a cell viability assay. Cell viability is expressed as a percentage relative to untreated control cells. Data are presented as mean ± standard deviation (n = 5).


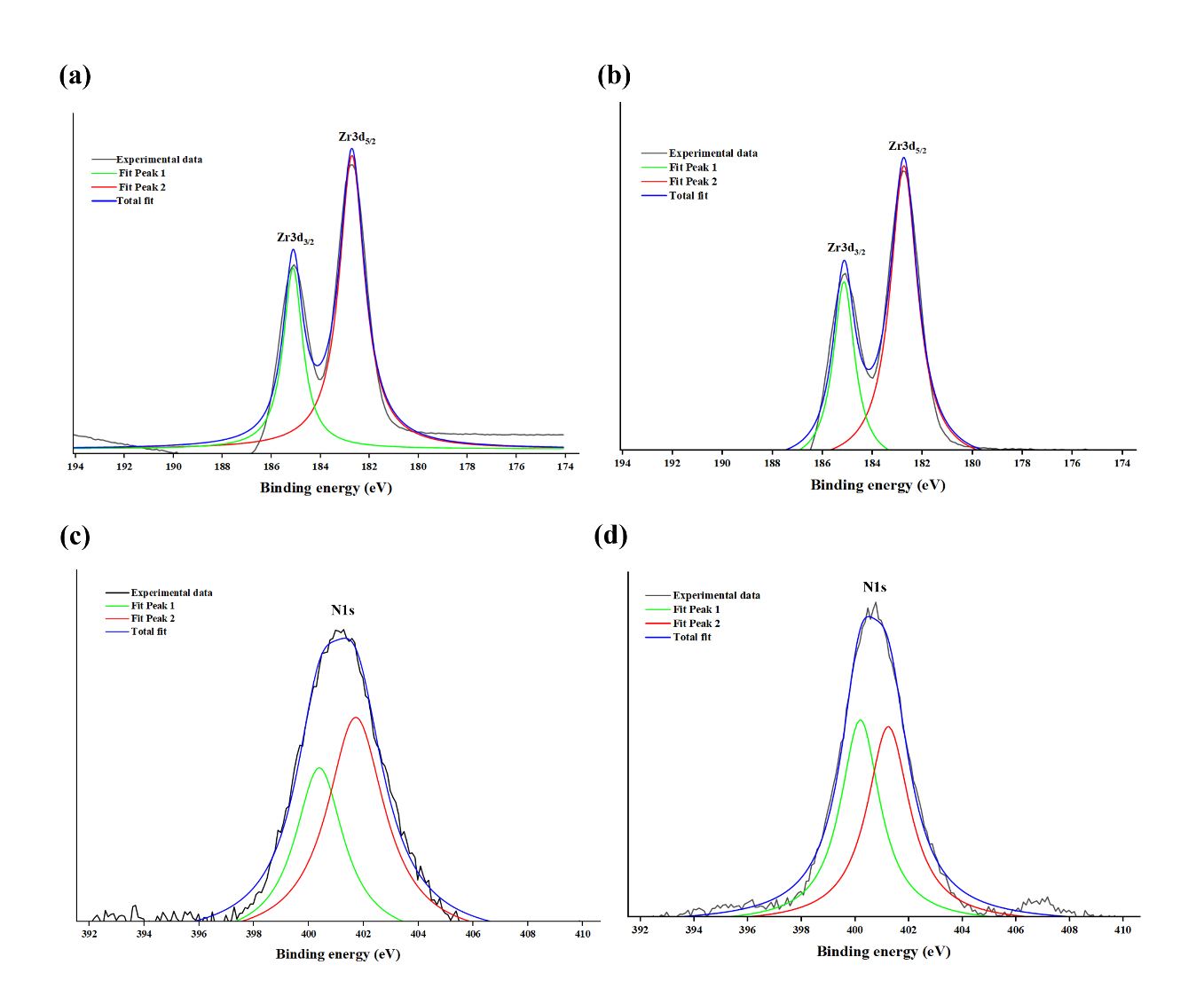


Figure S13. High resolution Zr 3d XPS spectra and deconvolution results of (a) MIP-202 and (b) AIM-202, together with high resolution N 1s XPS spectra and deconvolution results of (c) MIP-202 and (d) AIM-202. The experimental spectra are shown in black, the individual fitted components are shown in green and red, and the overall fitted envelopes are shown in blue. For the Zr 3d spectra, the fitted peaks correspond to the spin orbit doublet of Zr 3d₅/₂ and Zr 3d₃/₂. The similar binding energy positions and fitting profiles of MIP-202 and AIM-202 indicate that the Zr chemical state is largely preserved after Ag incorporation, with no clear evidence for the formation of additional Zr species. For the N 1s spectra, the experimental envelopes were deconvoluted into two fitted components. The comparable peak positions and fitting profiles suggest that the nitrogen chemical environment remains similar in both samples.

Table S4. Comparison of quenching models for Cu²⁺ quantification. Summary of the Langmuir quenching model, PET-derived quenching model, and AI-enhanced quenching model, including their equations, implementation, required parameters, fit quality, applicable ranges, and recommended uses.

| **Feature** | **Langmuir Quenching Model** | **PET-Derived Quenching Model** | **AI-Enhanced Quenching Model** |
| --- | --- | --- | --- |
| **Model type** | Mechanistic  (Langmuir isotherm-based) | Mechanistic + empirical (Langmuir-quadratic hybrid) | Hybrid (Mechanistic + SVR-based residual correction) |
| **Equation** | $F\left( Q \right)=\frac{F_{0}}{\left( 1+K\cdot Q \right)}$ | $F\left( Q \right)=F_{0}\cdot\left( 1-\frac{K\cdot Q}{1+K\cdot Q} \right)+a{\cdot Q}^{2}+b\cdot Q^{2}$ | $F_{final}\left( Q \right)=F_{base}\left( Q \right)+SVR(Q)$ |
| **Implementation** | Closed-form analytical expression | Closed-form with additional nonlinear terms | Python-based (scikit-learn, SVR, curve fitting, Jupyter Notebook) |
| **Required parameters** | F_0_, K | F_0_, K, a, b | F_0_, K, a, b + SVR model parameters (kernel, γ, C, ε) |
| **Fit quality (R²)** | 0.9354 | 0.9909 | 0.9999 |
| **RMSE** | 33739.08 | 12649.73 | 94.01 |
| **Applicable range** | Limited to low-to-moderate Q (no saturation correction) | Broader, captures saturation and curvature | Very broad, adapts to complex nonlinear deviations across the full range |
| **Recommended use** | Simple mechanistic fitting and theoretical analysis | Lab-based quantification, publication-ready modeling | High-precision field applications, adaptive sensing platforms |

Table S5. Comparison of the quenching model for Fe^3+^ quantification. Summary of the Stern–Volmer quenching model, PET-derived quenching model, and AI-enhanced quenching model, including their equations, implementation, required parameters, fit quality, applicable ranges, and recommended uses.

| **Feature** | **Stern–Volmer**  **Quenching Model** | **PET-Derived Quenching Model** | **AI-Enhanced Quenching Model** |
| --- | --- | --- | --- |
| **Model type** | Mechanistic  (Linear Stern–Volmer) | Mechanistic  (Log-extended Stern–Volmer) | Hybrid  (Logarithmic + AI residuals) |
| **Equation** | $\begin{aligned} \frac{F_{0}}{F}=1+K_{SV}\left[ Q \right] \end{aligned}$ | $F\left( Q \right)=F_{0}\cdot\left( 1-\alpha\cdot{log}_{e} \left( 1+\gamma Q \right) \right)$ | $F\left( Q \right)=F0\cdot\left( 1-\alpha\cdot\ln\left( 1+\delta Q \right) \right)+aQ+bQ2+cQ3$ |
| **Implementation** | Closed-form analytical expression | Closed-form analytical expression | Python (scikit-learn, Jupyter Notebook) |
| **Required parameters** | F₀, K_SV_ | F₀, α (quenching efficiency), γ (sensitivity) | F₀, α, γ + Polynomial regression coefficients |
| **Fit quality (R²)** | 0.9537 | 0.98667 | 0.9962 |
|  |  |  |  |
| **RMSE** | 37300.04 | 17438.04 | 10113.63 |
| **Applicable range** | Narrow, best for low concentration, linear region | Broad, includes nonlinear and saturation regions | Very broad, corrects deviations beyond saturation |
| **Recommended use** | Basic mechanistic interpretation, linear fitting | Mechanistic analysis, scientific reporting | Field applications, enhanced sensing precision |

**Table S6.** Training and test performance of AI-enhanced quenching models (LOOCV) and full-dataset refit metrics.

| **Ion** | **Evaluation scheme** | **Train split** | **Test split** | **R²** | **RMSE (a.u.)** |
| --- | --- | --- | --- | --- | --- |
| **Cu²⁺** | Full-dataset refit | 7/7 | 0/7 | 0.9999 | 94.01 |
| **Cu²⁺** | LOOCV (mean train) | 6/7 | 1/7 | 0.9999 | 165.34 |
| **Cu²⁺** | LOOCV (test) | 6/7 | 1/7 | 0.9494 | 29865.31 |
| **Fe³⁺** | Full-dataset refit | 7/7 | 0/7 | 0.9962 | 10113.63 |
| **Fe³⁺** | LOOCV (mean train) | 6/7 | 1/7 | 0.9964 | 9385.55 |
| **Fe³⁺** | LOOCV (test) | 6/7 | 1/7 | 0.9034 | 50819.39 |

**Table S7.** Comparison of photocatalytic performance and functional integration of AIM-202 with previously reported Ag-MOF, Zr-MOF, and plastic degradation systems.

| **Catalyst System** | **Target Substrate** | **Reaction Conditions** | **Photocatalytic Outcome** | **Sensing Integration** | **Ref** |
| --- | --- | --- | --- | --- | --- |
| Zr-MOF  (UiO-66–derived) | PET powder | High temperature (260 °C), methanol | Glycolysis to BHET (>90% yield) | No (chemical recycling only) | [1] |
| Ag-MOF (Ag/AgCl@MIL-88A) | Organic dyes / phenols | Sunlight, RT, water | Photodegradation of dissolved pollutants | No (degradation only) | [2] |
| CdS/CdOₓ quantum dots | PET / PLA plastics | Visible light, RT, alkaline conditions | H₂ production (waste-to-energy conversion) | No (fuel production only) | [3] |
| **AIM-202  (This work)** | PET microplastics (granules) | Xe lamp (visible light), RT, water | Depolymerization to TPA / 2-HTPA (28.7% yield) | Yes (in situ–generated) | This work |

1. Wu Y, Wang X, Kirlikovali KO, Gong X, Atilgan A, Ma K, Schweitzer NM, Gianneschi NC, Li Z, Zhang X: **Catalytic degradation of polyethylene terephthalate using a phase‐transitional zirconium‐based metal–organic framework.** *Angewandte Chemie International Edition* 2022, **61:**e202117528.

2. Viswanathan VP, Divya K, Dubal DP, Adarsh NN, Mathew S: **Ag/AgCl@ MIL-88A (Fe) heterojunction ternary composites: towards the photocatalytic degradation of organic pollutants.** *Dalton Transactions* 2021, **50:**2891–2902.

3. Uekert T, Kuehnel MF, Wakerley DW, Reisner E: **Plastic waste as a feedstock for solar-driven H 2 generation.** *Energy & Environmental Science* 2018, **11:**2853–2857.
